# Proof-of-concept study: *APOE4* brain endothelial cells as a phenotypic compound screen

**DOI:** 10.1186/s13195-026-01960-6

**Published:** 2026-02-02

**Authors:** Ana C. Valencia-Olvera, Felecia M Marottoli, Kiira Ratia, Gregory RJ Thatcher, Leon Maing Tai

**Affiliations:** 1 Department of Anatomy and Cell Biology, University of Illinois, M/C 512, Rm 578, 808 S. Wood St, Chicago, IL 60612 USA; 2 Department of Pharmaceutical Sciences, University of Illinois College of Pharmacy, University of Illinois at Chicago, Chicago, IL USA; 3 Dept of Pharmacology & Toxicology, University of Arizona, Tucson, AZ USA

**Keywords:** APOE4, Brain endothelial cells, Compound screen

## Abstract

**Background:**

Published data suggest that compared to *APOE3*, *APOE4* could increase the risk of neurodegeneration via higher cerebrovascular permeability. We recently proposed the concept that brain endothelial cell *APOE* is protective for cerebrovascular function in a genotype specific manner, *APOE3* > *APOE4*, and therefore *APOE4* brain endothelial cells may be predisposed to dysfunction during aging and disease. In addition to mechanistic implications, our concepts and methods may have therapeutic applications; identifying compounds that protect *APOE4* brain endothelial cells. The goal of this proof-of-concept study was to determine whether *APOE4* brain endothelial cells can be used as a phenotypic compound screen.

**Methods:**

Previously we found that *APOE4* brain endothelial cells are particularly sensitive to lipopolysaccharide- (LPS) induced permeability disruption when measured by trans endothelial cell electrical resistance (TEER) in vitro. Here, we followed the NIH Assay guidance manual to convert our in vitro assay to a phenotypic screen. We scaled the isolation protocol, selected conditions for the min, mid and max signals, statistically validated the phenotypic assay, screened compounds, validated hits and tested the top hits in vivo.

**Results:**

We scaled the isolation protocol and selected conditions for min (0.8 µg/ml LPS), mid (10 µM sildenafil/LPS) and max conditions (vehicle). Our final protocol met the reproducibility acceptance criteria for a statistically validated assay. We then screened a subset of ~ 900 molecules from the TargetMol Bioactive Library and identified two main groups compounds. The first group disrupted *APOE4* brain endothelial cells as they were toxic or lowered TEER and many inhibited mTOR. The second group protected against LPS-induced TEER reduction. With relatively stringent criteria we identified 33 protective compounds that are grouped into those that inhibit growth factor receptor signaling and a range of intracellular signaling pathways. We compared the most active compounds and selected four to test in vivo. Tadalafil (PDE5 inhibitor), vorinostat (HDAC inhibitor), CCT196969 (raf inhibitor) and SGI-7079 (AXL inhibitor) mitigated acute LPS-induced cerebrovascular dysfunction in mice that express *APOE4*.

**Conclusions:**

Overall, our data supports the potential of our in vitro screen to identify compounds that prevent LPS-induced dysfunction in *APOE4* brain endothelial cells.

**Supplementary Information:**

The online version contains supplementary material available at 10.1186/s13195-026-01960-6.

## Introduction

*APOE* is a major genetic risk factor for several age-related neurodegenerative disorders. Compared to *APOE3*, *APOE4* is associated with greater cognitive dysfunction in older adults, increased Alzheimer’s disease risk and exacerbated progression of vascular dementia, stroke, and traumatic brain injury [1–4]. Based on these clinical data, research continues to identify functions that are affected by *APOE* genotypes in aging and neurodegenerative contexts [5]. Through these studies, it has emerged that *APOE* genotype could contribute to altered neuron function via modulation of the cerebrovasculature [6, 7]. Markers of cerebrovascular disruption including higher leakiness, vessel degeneration, changes in cerebral blood flow and vascular inflammation are greater with *APOE4* compared to *APOE3* during aging, in Alzheimer’s disease and in respective mouse models [6, 8–17]. Higher cerebrovascular leakiness is considered particularly detrimental for brain function, through disrupting the neuronal environment and via entry of proteins and other toxins that can damage neurons directly and via effects on supporting cells. Thus, identifying therapeutic approaches that mitigate *APOE4* associated cerebrovascular dysfunction is a key approach to address neurodegenerative disorders.

Specialized brain endothelial cells are the major effector cells of the cerebrovasculature and control the bi-direction movement of essential and unwanted molecules to and from the brain. Mechanistically, research has focused on how the *APOE* genotype of non-brain endothelial cells regulates cerebrovascular function [12, 13]. We recently identified an additional pathway that brain endothelial *APOE* regulates neural function. Supported by our in vitro and in vivo data we proposed the concept that brain endothelial cell *APOE* is protective for cerebrovascular function in a genotype specific manner, *APOE3* > *APOE4*, and therefore *APOE4* brain endothelial cells may be predisposed to dysfunction during aging and disease [18–20]. Testing that mechanism is the focus of our planned studies, however our concepts and assays may also have therapeutic applications; identifying compounds that protect *APOE4* brain endothelial cells. Our in vitro isolation protocols and readout of permeability (trans endothelial cell electrical resistance; TEER) may be scalable to allow screening. Further, we have identified in a pilot study that *APOE4* brain endothelial cells are particularly sensitive to inflammation- induced (lipopolysaccharide; LPS) TEER disruption [18]. Those data are consistent with findings that *APOE4* interacts with neuroinflammation and peripheral inflammation to induce brain endothelial cell dysfunction in vivo and increase dementia risk/progression [21–30]. Therefore, our in vitro model may provide an opportunity to determine the extent that *APOE4* brain endothelial cells can be used to identify protective compounds.

The goal of this proof-of-concept study was to determine whether *APOE4* brain endothelial cells can be used as a phenotypic compound screen. To that end, we optimized and statistically validated our in vitro model of LPS-induced paracellular permeability disruption (TEER) in *APOE4* brain endothelial cells. We then screened 900 + mainly FDA approved compounds and tested the top 4 in vivo using an acute model of LPS-induced cerebrovascular permeability in *APOE4*-knock in mice.

## Methods

### Mice

Experiments were approved by the Institutional Animal Care and Use Committee at the University of Illinois at Chicago. We used mice that express human *APOE4* under the endogenous mouse *APOE* promoter (EFAD-; [25, 31, 32]. Mice were housed on a 12 h light/dark cycle with food (Teklad 7912 from Inotiv) and water ad libitum. For brain endothelial cell isolation, mice were weaned in 4–5 groups for a week or weaned right before isolation. For the in vivo study, mice were grouped in cages of 4–5 mice.

### Brain endothelial cell isolation and trans-endothelial electrical resistance

We have previously published our step-by-step primary mouse brain endothelial cell isolation protocol [18]. That version was optimized for smaller scale mechanistic isolations in which one to four mice represent a single biological *n* depending on the growth surface size. For this project we made a few amendments to increase scale. Therefore, here we provide a brief description of the protocol and highlight the differences.

Cerebral cortices were dissected from 21-28-day-old male and female mice, diced, centrifuged, resuspended in a digest buffer containing papain (20 U/ml)/DNase (2,000 U/ml) and triturated through a 19G needle. For each 96 well plate, cells isolated from 16 to 18 mice were pooled prior to plating. To achieve that, after dissection, the 16–18 mice were split into two groups of 8–9 mice and each pellet was resuspended in 5.5 ml of digest buffer each. They were then homogenized using a needle much more vigorously than for single brain isolations to fully break up the tissue. The tubes were processed separately and then pooled and mixed in the final step. Homogenates were then incubated for 15 min at 37 °C, triturated (21G), mixed in 2 x volume of 25% BSA, vortexed and centrifuged (4,000x g, 5 min, 4 °C). The pellet was resuspended in heparin-supplemented (5.5 U/ml heparin) EBM-2 media (containing EGMTM-2 MV Microvascular Endothelial SingleQuotsTM). The supernatant was collected, vortexed, and centrifuged a second time (4,000x g, 5 min, 4 °C). Pellets from both centrifugations were combined and passed through a 100 μm cell strainer and pelleted (1,000 x g, 5 min, 4 °C). Cells were resuspended with EBM-2 media + heparin and plated on 96w10idf plates (Applied Biophysics) that had been coated with a growth matrix of fibronectin, collagen and laminin. 16 h after plating, red blood cells and debris were washed away, which is longer than for single brains (6 h), as in our initial experiments we found the cells were less firmly attached than single brain isolations. Cells were then incubated in 8 mg/ml puromycin (EBM-2 media) for 48 h. Our isolation protocol is based on negative selection using puromycin, since brain endothelial cells express high levels of efflux transporters (e.g. p-glycoprotein) which prevents toxicity. After puromycin was removed, brain endothelial cells were washed and grown to confluence in EBM-2 media for 72 h.

**Fig. 1:**
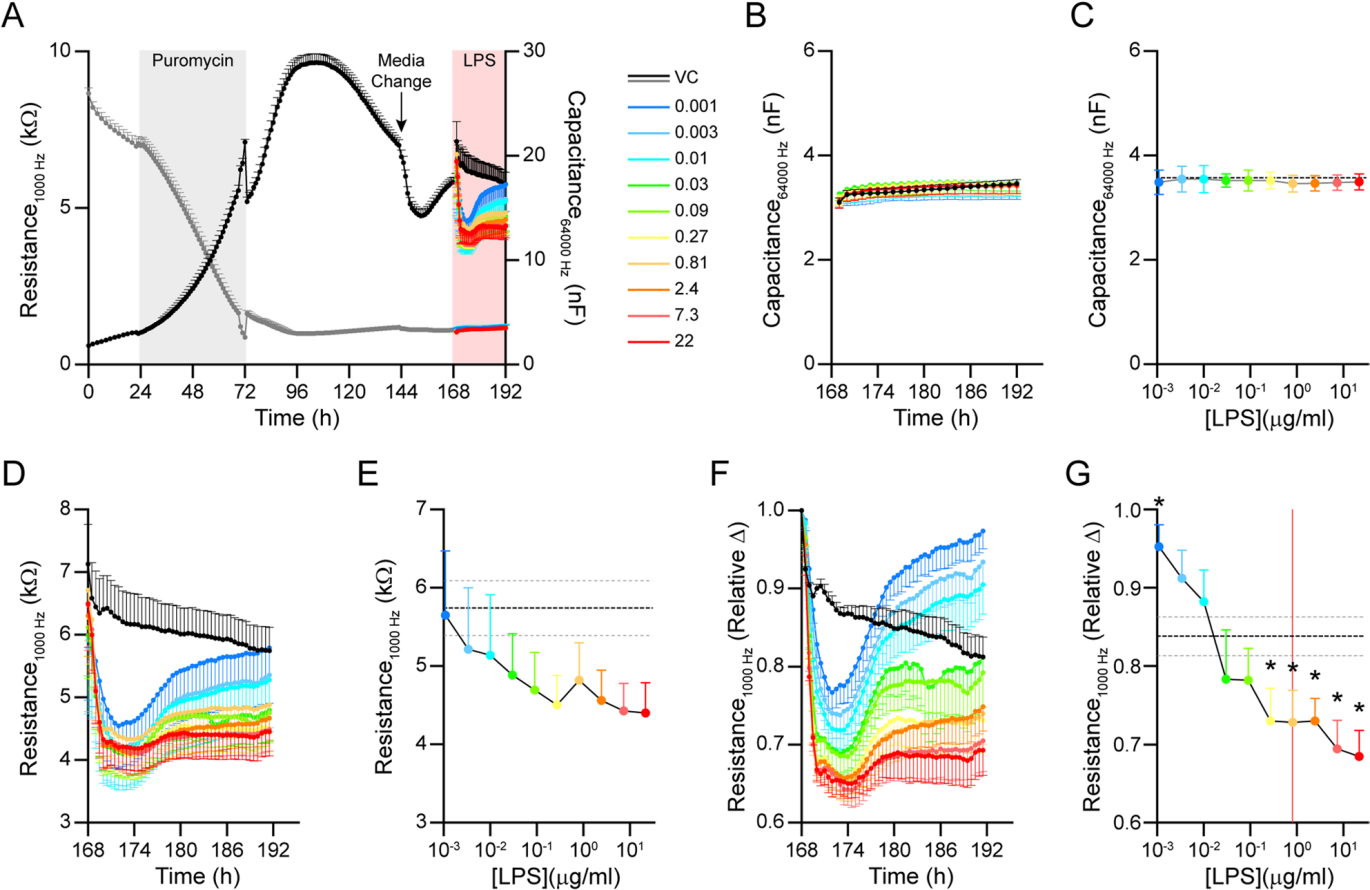
Selecting an LPS concentration for the min signal. *APOE4* brain endothelial cells were treated for 24 h with LPS (0.004–22 µg/ml) to select a concentration of LPS that produced sustained permeability disruption but was non-toxic. **A** Following isolation, *APOE4* brain endothelial cells established a confluent monolayer after 72 h, evident by low capacitance (64 kHz; grey lines) and maximum TEER value (1000 Hz, black lines). After reaching a plateau there was a slow decline in TEER but not capacitance until steady state was reached (~ 120 h) and the cells were sensitive to full media changes but recovered and remained stable. **B**, **C**. LPS treatment (0.004–22 µg/ml) had no impact on capacitance. **B** Represents capacitance over time with LPS treatment and (**C**) is capacitance at 18 h with LPS treatment. After LPS treatment, TEER was evaluated in two ways. The first was raw resistance values (**D**, **E**) and the second relative change (**F**, **G**) which is TEER values pre-LPS/post-LPS. D-F represents time course changes in TEER with LPS and E-G TEER at 18 h. Between the two evaluation methods, relative change was selected for the rest of the screen as it accounts for variations in baseline TEER within each well. When evaluated by relative change at 18 h there was an effect of LPS treatment (F_(10, 27)_ = 12.23, *p* < 0.0001), with post hoc revealing that compared to vehicle, TEER levels were higher at 0.001 µg/ml LPS (*p* = 0.0297) and lower at 0.3 µg/ml (*p* = 0.0225), 0.8 µg/ml (*p* = 0.0195), 2.4 µg/ml (*p* = 0.0226), 7.3 µg/ml (*p* = 0.0015), and 22 µg/ml (*p* = 0.0007) LPS. Data analyzed by matched Mixed-effects model (REML) followed by Dunnet’s multiple comparisons test comparing each LPS concentration to the vehicle. *p < 0.05 for a given LPS concentration compared to vehicle control in G. *n* = 4, when an *n* represents a different isolation. All statistical analysis is provided in Additional File 2

**Fig. 2:**
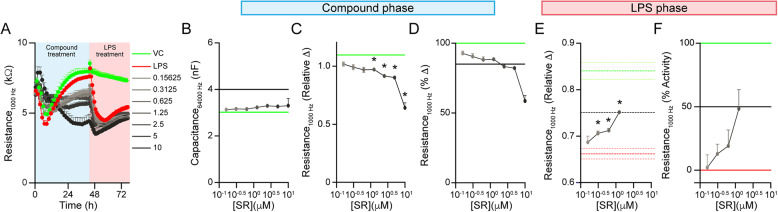
Evaluation of SR9009 as the mid signal.** A ***APOE4* brain endothelial cells were treated with media containing SR9009 (0.15-10 µM) for 24 h, and then 0.8 µg/ml LPS was spiked in for a further 18 h. **B**-**D** SR9009 treatment phase. **B** SR9009 treatment was nontoxic (capacitance < 4nF). **C** For relative change, there was an effect of SR9009 treatment (F_(1.538, 3.077)_ = 41.80, *p* = 0.0062) driven by lower values at 10 µM (*p* = 0.0291); 5 µM (*p* = 0.0108); 2.5 µM (*p* = 0.0115) and 1.25 µM (*p* = 0.0082) compared to vehicle. **D** When evaluated as percent change, SR9009 reduced TEER by at least 15% at concentrations of 2.5–10 µM. Thus, concentrations below 1.25 µM were only included for the LPS treatment phase. **E**, **F** LPS treatment phase. **E** When evaluated as relative change, there was an effect of SR9009 treatment (F_(4, 8)_ = 13.28, *p* = 0.0013) as values were higher for 1.25 µM (*p* = 0.0004); 0.63 µM (*p* = 0.0198); and 0.31 µM (*p* = 0.0428) SR9009 treatment compared to LPS control. **F** When evaluated as percent activity (Vehicle: 100% and LPS: 0%), only 1.25 µM reached 50%. Data analyzed by repeated measured one-way ANOVA, followed by Dunnet’s multiple comparisons test comparing a compound concentration to the control group. In the compound treatment phase (**B** and **C**), *p < 0.05 for a given SR9009 concentration compared to vehicle control. In the LPS treatment phase (**E**), *p < 0.05 for a given SR9009 concentration + LPS compared to the vehicle + LPS (LPS control). *n* = 3 All statistical analysis is provided in Additional File 2

**Fig. 3:**
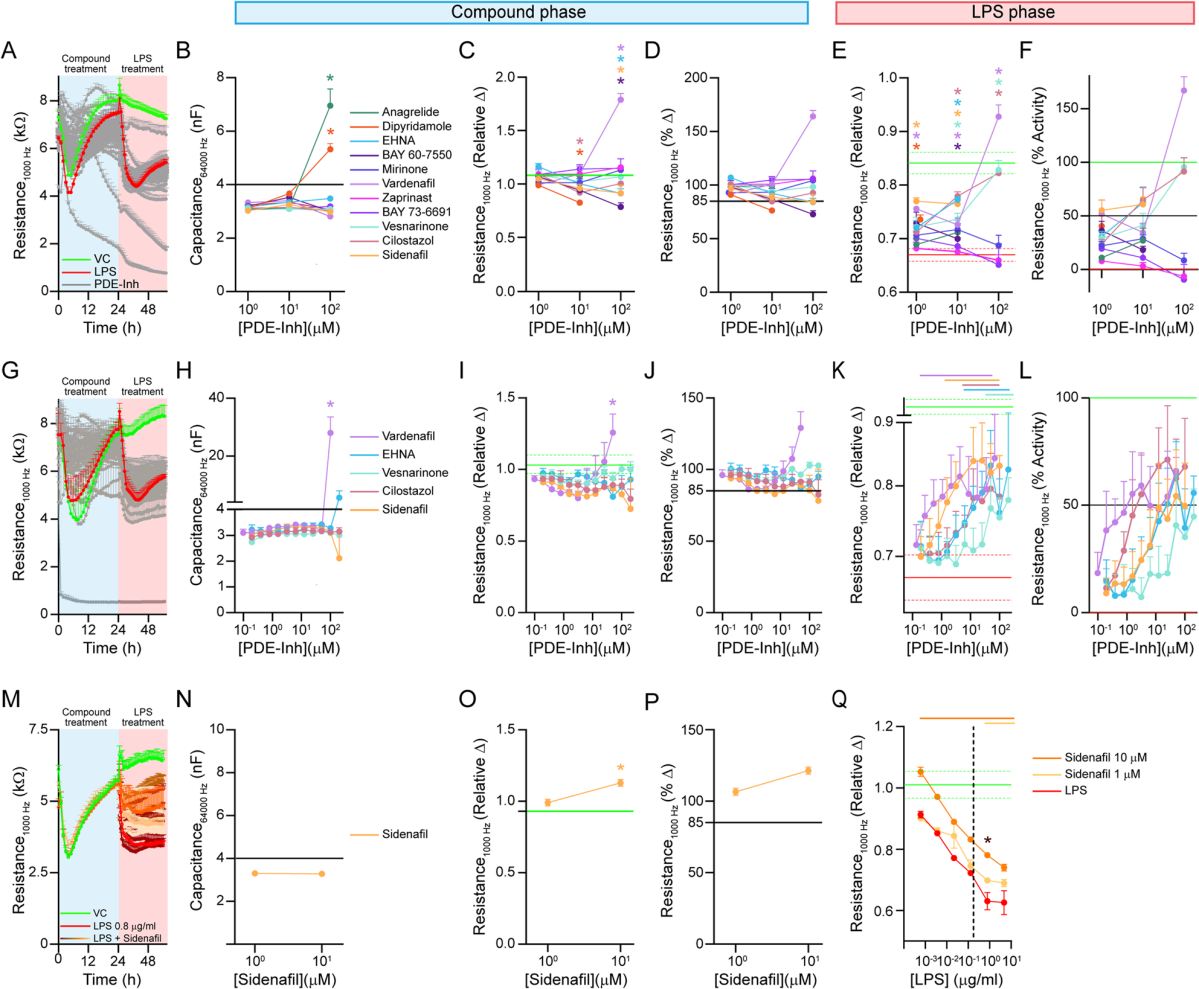
Evaluation of phosphodiesterase inhibitors as the mid signal. Inhibitors of phosphodiesterase (PDE) 2 (EHNA, BAY60-7550), PDE3 (cilostazol, milrinone, anagrelide, vesnarinone), PDE5 (dipyridamole, sildenafil, vardenafil, zaprinast) and PDE9A (BAY73-6691) were evaluated for their ability to mitigate LPS-induced TEER reduction in *APOE4* brain endothelial cells. **A**-**F** An initial screen was conducted with each compound at 1, 10 and 100 µM. **B**-**D** Compound treatment phase. Compound concentrations were removed that **B**. had capacitance > 4nF (100 µM anagrelide and dipyridamole) and **C**. lowered relative TEER compared to vehicle and met our cut off criteria of a 15% decrease (**D**, 10 µM dipyridamole, 100 µM BAY60-7550, EHNA and sildenafil). **E**, **F**. LPS phase. Vardenafil, vesnarinone, cilostazol and sildenafil mitigated LPS-induced relative TEER disruption for at least 2 concentrations and met our 50% activity criteria. **G**-**L** Expanded concentration-response (0.2–200 µM) evaluation of vardenafil, sidenafil, vesnarinone, cilostazol and EHNA. **H**-**J** Compound treatment phase. **H** 100 µM vardenafil was toxic (capacitance > 4nF). **I**, **J** 50 µM vardenafil increased baseline TEER and 200 µM cilostazol and sildenafil disrupted TEER (% change < 85%). LPS phase. For relative change, vardenafil (0.4–50 µM), sildenafil (1.56–100 µM), EHNA/cilostazol (12.5–200 µM) and vesnarinone (200 and 50 µM) reduced LPS-induced TEER reduction (colored bars represent concentrations that were significantly different compared to LPS control). When assessed by percent activity, concentrations that met the 50% cut-off were: cilostazol (≥ 50 µM), EHNA (≥ 25 µM), sildenafil (≥ 3.125 µM) and vardenafil (≥ 1.5625 µM). **M**-**Q** LPS-induced TEER reduction as modified by Sidenafil. **N**-**P** Compound treatment phase. Sidenafil 10 µM increased baseline relative change in TEER. **Q** LPS phase. Sidenafil at 10 µM protected from all LPS concentrations tested while 1 µM only protected at 5 and 0.8 µg/ml LPS. Only at 0.8 µg/ml of LPS there was a difference between the 3 groups (LPS, LPS + Sil 10 µM, and LPS + Sil 1 µM). Data analyzed by one-way ANOVA/matched Mixed-effects model (REML) followed by Dunnet’s multiple comparisons test: in the compound treatment phase (**B**, **C**, **H**, **I**, and **N**-**P**), *p < 0.05 for a given compound concentration compared to vehicle. In the LPS treatment phase (**E**, **K**, **Q**), colored line: *p* < 0.05 for a given compound concentration + LPS compared to the vehicle + LPS (LPS control). In Q: *p < 0.05 for 10 µM vs. 1 µM Sidenafil. *n* = 3. All statistical analysis is provided in Additional File 2

**Fig. 4:**
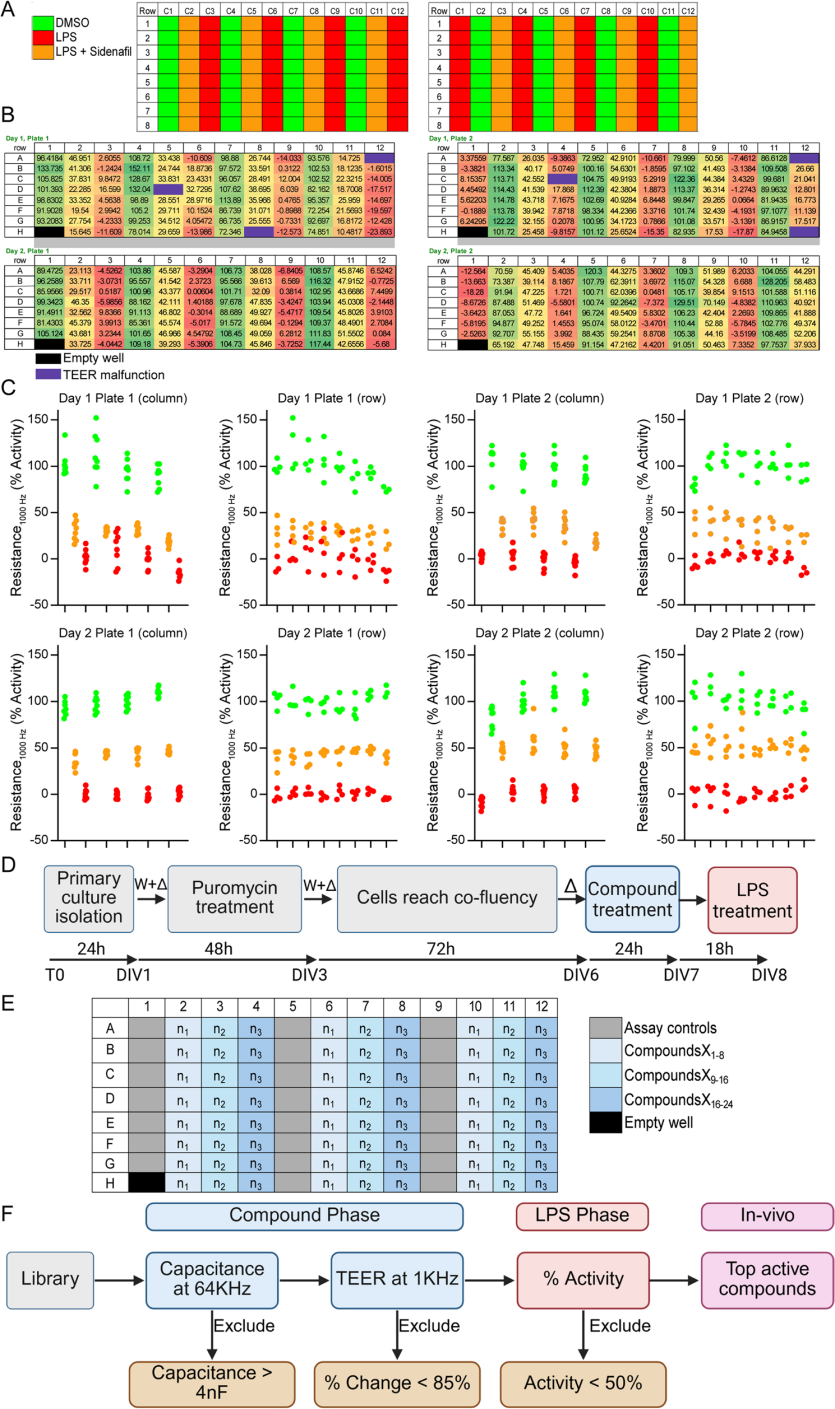
Phenotypic screen statistic validation. Evaluation of the *APOE4* brain endothelial cells assay as a phenotypic drug screen according to the NIH Assay guidance manual. **A**, **B** The reproducibility of the max (water, DMSO), min (LPS, DMSO) and mid-signal (LPS, Sidenafil) were evaluated using the interleaved format of two plates on two different days. **C** Scatter plots of the resulting data showed no edge effects or drift across plates and between days. *n* = 4 The data presented in **C**. was used for the calculations in Table 2. **D ***APOE4*-endothelial cells primary culture workflow, w: washing cells, Δ: Media change. **E** Compound screen plate layout. Each plate screened 24 compounds in triplicate. **F** Compound screen workflow with exclusion criteria

**Fig. 5:**
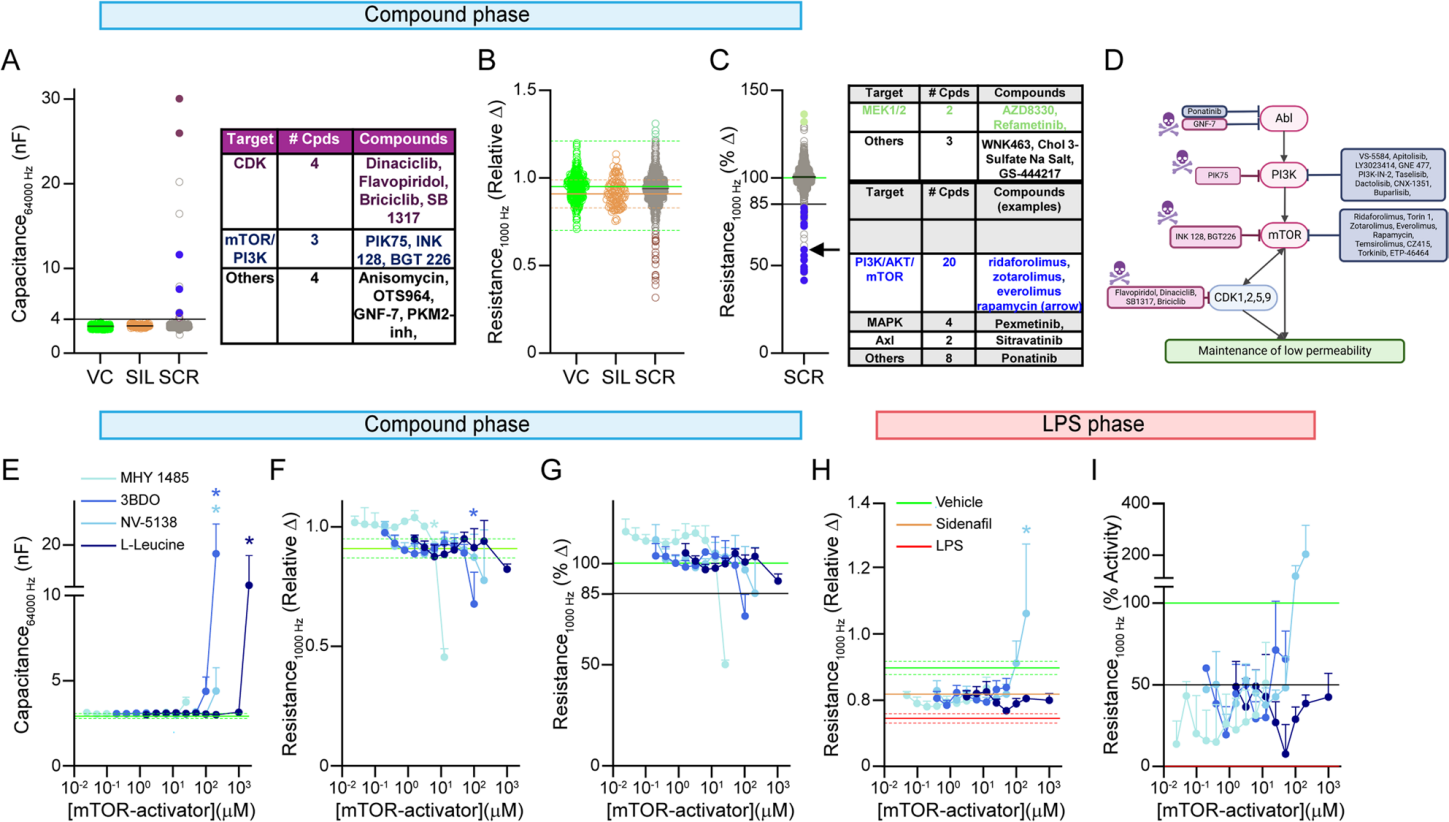
Compound treatment phase, mTOR inhibitors disrupt baseline TEER of *APOE4*-brain endothelial cells. *APOE4*-brain endothelial cells were treated for 24 h with 1 µM of 900 + compounds selected from the TargetMol Bioactive Library. **A** Capacitance was > 4nF for 11 compounds, 4 of which inhibit cell cycle pathways (CDK, purple dots) and 3 PI3K/Akt/mTOR (blue dots). **B**, **C** Relative change and percentage activity of compounds. We identified compounds that either increased (light green dots, 5 compounds) or decreased (brown dots, 34 compounds) baseline TEER. 22 were inhibitors of mTOR/PI3K (blue dots) including rapamycin (arrow). **E**-**I** The ability of mTOR activators (NV-5138, L-leucine, 3BDO, MHY1485) to modulate LPS-induced TEER disruption was evaluated. **E**, **G** Compound treatment phase. E. High concentrations of mTOR activators were either toxic (2 mM L-leucine; 200 µM 3BDO) or F. disrupted TEER (100 µM 3BDO). **H**, **I** LPS Phase. High concentrations of NV-5138 (10 µM) mitigated LPS-induced TEER disruption. Data analyzed by one-Way ANOVA/matched Mixed-effects model (REML) followed by Dunnet’s multiple comparisons test comparing a compound concentration to the control group. In the compound treatment phase (**F**), *p < 0.05 for a given compound concentration compared to vehicle. In the LPS treatment phase (**H**), *p < 0.05 for a given compound concentration + LPS compared to the vehicle plus LPS group. *n* = 3. Upper lines indicate the concentrations different from LPS control for each compound. All statistical analysis is provided in Additional File 2

**Fig. 6:**
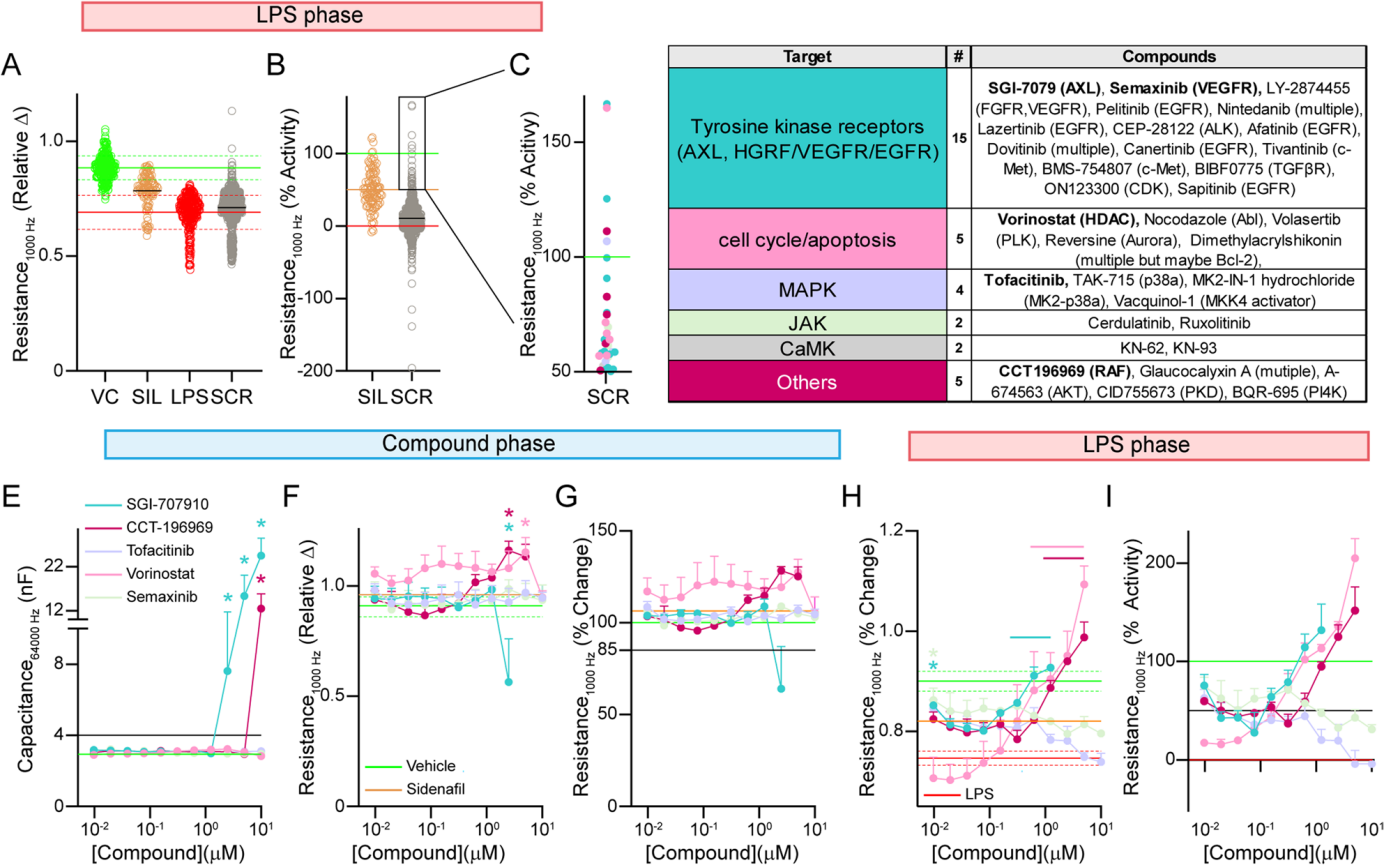
LPS phase, 33 active compounds prevent LPS-induced TEER disruption.** A**-**C** Relative change and percentage activity of 860 compounds that passed the criteria in the compound treatment phase. Among the active compounds, 33 compounds show activity greater than 50% and include inhibitors of growth factor signaling (15 compounds, blue dots), the cell cycle/apoptosis (5 compounds, maroon dots), and MAPK/JAK/CaMK (4 compounds, teal dots). The most potent compounds were SGI-7079 (AXL inhibitor), vorinostat (HDAC inhibitor), semaxinib (VEGFR inhibitor), CCT196969 (raf inhibitor) and tofacitinib (Jak inhibitor), which we validated with concentration response curves in (**E**-**I**).** E**-**G** Compound phase. **E** Toxic compound concentrations were SGI-7079 (≥ 5 µM) and CCT196969 (10 µM). **G **SGI-7079 also disrupted TEER by greater than 15% at 2.5 µM. CCT196969 (5 µM and 2.5 µM) and vorinostat at (5 µM) treatment resulted in higher baseline relative TEER values. **H**, **I** LPS Phase. Vorinostat (0.625-10 µM), CCT-196,969 (1.25-5 µM) and SGI-7079 (0.3158-1.25 µM) mitigated LPS-induced TEER reduction. Compounds that met the 50% activity criteria were SGI-7079 (0.01, 0.156–1.25 µM); vorinostat (0.13-5 µM); Semaxinib (0.01–0.625 µM); Tofacitinib (0.01 µM); and CCT-196,969 (0.313-5 µM). Data analyzed by matched Mixed-effects model (REML) followed by Dunnet’s multiple comparisons test. *p < 0.05. *n* = 3. Upper lines indicate the concentrations different from LPS control for each compound. All statistical analysis is provided in Additional File 2

**Fig. 7:**
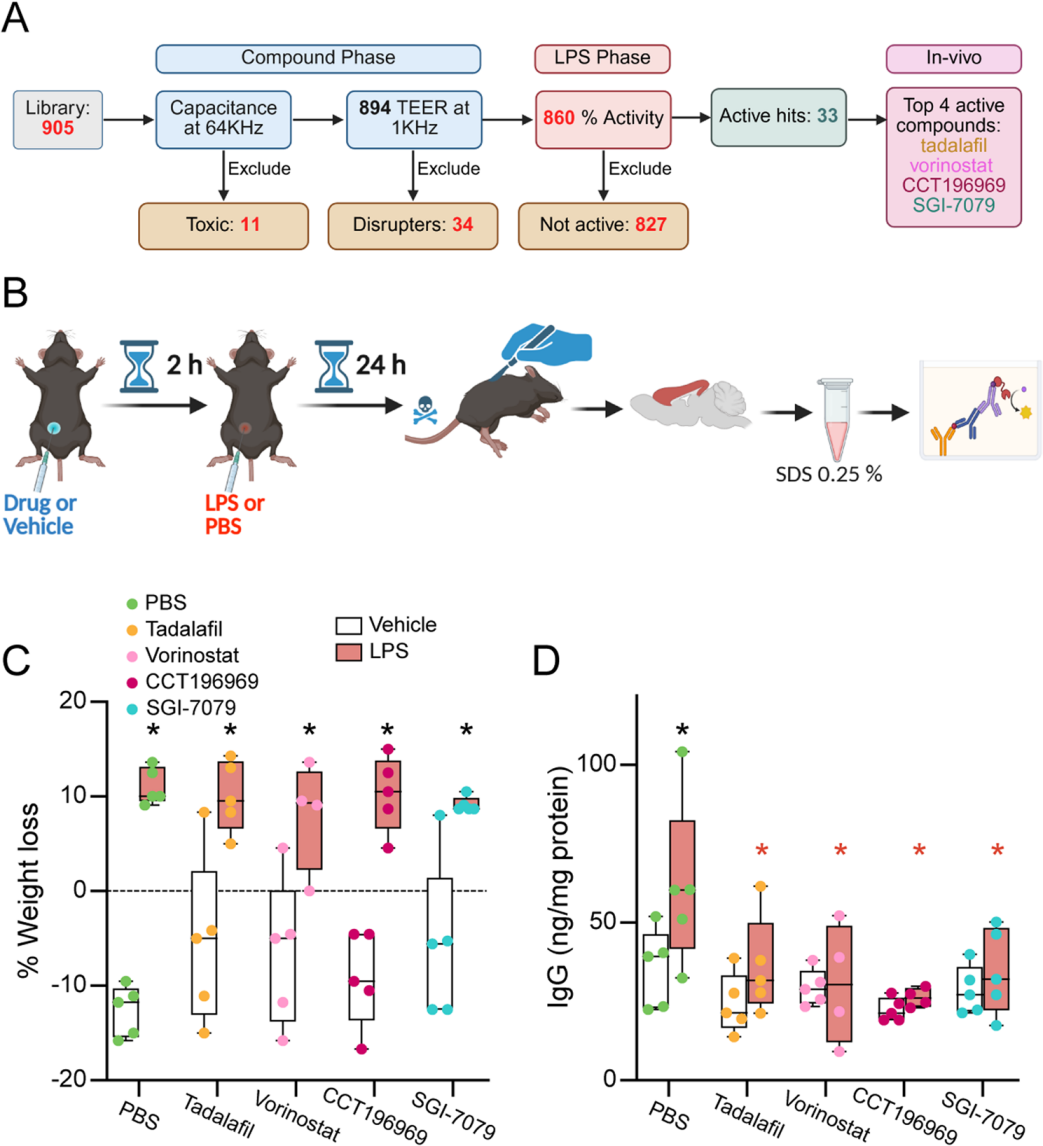
Compounds identified in the HTS screen prevent acute LPS-induced plasma proteins brain permeability in vivo. **A** Summary of phenotypic screen data. Compound treatment phase. *APOE4* brain endothelial cells were treated with 905 molecules for 24 h. 11 compounds were toxic to brain endothelial cells and 34 reduced trans-endothelial electric resistance (TEER) bellow 85%. LPS treatment phase. We identified 33 compounds that mitigated LPS-induced TEER reduction with 50% activity or greater. We selected three to test in vivo along with a positive control compound (tadalafil). **B** In vivo study design. 5-month-old female *APOE4* knock in mice were treated (i.p.) with either vehicle (5% DMSO and 1% Tween 80 in saline), tadalafil (20 mg/kg), vorinostat (50 mg/kg), CCT196969 (20 mg/kg) or SGI-7079 (50 mg/kg) for 2 h and then 24 h with either PBS or LPS (1 mg/ml) treatment (i.p.). Cortices were homogenized (0.25% SDS) and IgG levels were measured by ELISA and normalized to protein concentrations. **C** LPS (F _(1, 39)_ = 122.1, *p* < 0.0001) induced weight loss in all groups of mice. *p < 0.005. **D** For IgG levels, there was an effect of LPS (F_(1, 38)_ = 6.245, *p* = 0.0169) and compound treatment (F_(4, 38)_ = 4.488, *p* = 0.0046) but was not an interaction (F_(4, 38)_ = 1.307, *p* = 0.2848). However, as our main question was whether compounds could prevent LPS-induced leakiness, we performed post hoc analysis comparing all groups to the vehicle + LPS treated mice. We found that IgG levels were higher in vehicle + LPS compared to vehicle + PBS (black asterisk, *p* = 0.0041). In addition, IgG levels were lower in mice treated with any of the four compounds + LPS compared to vehicle + LPS (red asterisk; tadalafil *p* = 0.0366, vorinostat *p* = 0.012, CCT *p* = 0.003, and SGI *p* = 0.024). Data was analyzed by 2-way ANOVA followed by Tukey’s comparisons test. One outlier was detected by ROUT test (1%) at the group treated with CCT196969 + LPS, *n* = 5. All statistical analysis is provided in Additional File 2

Brain endothelial confluence was measured via capacitance (frequency of 64 K Hz) and permeability by trans endothelial electrical resistance (TEER, frequency of 10 K Hz) on an ECIS^®^ ZΘ (Applied Biophysics). For TEER, background resistance values (i.e., measurements from empty wells) were subtracted. In the compound treatment phase (see Results), fresh media (180 µl) containing compounds or vehicle were added for 24 h. All compounds were dissolved in DMSO except vardenafil. Therefore, DMSO is the vehicle for virtually all the manuscript. For concentrations-response experiments due to differences in compound solubility, DMSO concentrations varied across Figures. All compounds were buffer matched to the vehicle which corresponded to the following DMSO concentrations: 0.1% in Figs. 1, 2, 4, 5A-C, 6 and 7; 0.8% in Fig. 3; 0.3% in Fig. 5E-I. Vardenafil was dissolved in ethanol to a final in-well concentration of 5.6%. 24 h later, sterile lipopolysaccharide (LPS) from E. coli O8:K27 (S-form, innaxon) was spiked into the media and incubated for 18 h. In Figs. 2, 3, 4, 5, 6 and 7 the LPS stock of 1 mg/ml (in water) was diluted in EBM-2 media to a working concentration of 8 µg/ml (125-fold dilution) and then 20 µl was spiked into the 180 µl of media for a final concentration of 0.8 µg/ml. Therefore, the vehicle for LPS was EBM-2 media. In Supplementary Fig. 1 (Additional File 4) we also conducted a set of experiments where rapamycin was added at the same time as LPS, or 24 h after LPS.

### Western blot analysis

For western blot analysis *APOE4* brain endothelial cells were grown on fibronectin, collagen and laminin coated 12-well plates. The isolation procedure was the same as described in the section above, with the exception that one brain was isolated into one well of the 12-well plate. After the cells reached confluence, they were treated with rapamycin (1 µM) or vehicle for 24 h or vorinostat (1 µM) or vehicle for 24 h followed by vehicle or LPS (0.8 µg/ml) for an additional 24 h. Cells washed in TBS and lysed in 70 µl of RIPA buffer, sonicated (20% amplification, 3 cycles), centrifuged (25 000*g* for 20 min at 4 °C), flash frozen in liquid nitrogen and stored at − 80 °C. 7 µg of protein was loaded on 4% to 12% Bis-Tris Midi gels (Invitrogen) and separated by gel electrophoresis. Proteins were transferred onto low‐fluorescence PVDF membranes, which were cut, blocked (5% milk/TBS), and probed with primary antibody overnight at 4 °C (see Additional file 1) followed by the appropriate fluorescent secondary antibody (LI‐COR) for 45 min at room temperature. Proteins were imaged using the Odyssey Fc Imaging System and optical densities were quantified (Image Studio Lite v5.2).

### In vivo LPS study

All compounds injected into mice were prepared in a biosafety cabinet. Mice were weighed and treated with tadalafil (20 mg/kg) [33], CCT196969 (20 mg/kg) [34], SGI-7079 (50 mg/kg) [35] or vorinostat (50 mg/kg) [36]. All compounds were dissolved in 100% DMSO to appropriate stock concentrations (see Additional file 1) and then further suspended to working concentrations of 1.3 mg/ml (tadalafil & CCT196969), or 3.3 mg/ml (SGI-7079 & vorinostat) in saline containing 1% Tween 80. Thus, the final injected suspension contained 5% DMSO and 1% Tween 80 in saline which was used as a vehicle control. Injection volumes were based on bodyweight and were ~ 340 µl/mouse. 2 h after compound treatment, mice were treated with either 1 mg/kg LPS or vehicle (PBS). The LPS stock of 1 mg/ml (in water) was diluted in PBS to a working concentration of 0.2 mg/ml. Injection volumes were based on bodyweight and were ~ 120 µl/mouse.

24 h after LPS treatment, mice were anesthetized with ketamine (100 mg/kg) and xylazine (10 mg/kg) via intraperitoneal injection and transcardially perfused with 16 ml of TBS. Cortices were isolated and frozen in liquid nitrogen until processing. The tissue was homogenized using a bead mill (Fisherbrand) at 6 m/s for 1 × 30 s-cycle in SDS lysis buffer (0.25% SDS + 10 mmol/L NaF + 1× HALT protease inhibitor cocktail in 20 mmol/L HEPES; pH 7.4) at 15 µl/mg tissue. Samples were then centrifuged at 500*g* for 5 min at 4 °C, sonicated (20% amplification, 3 cycles), and then centrifuged again (25 000*g* for 20 min at 4 °C). Aliquots of the resulting supernatant were flash frozen in liquid nitrogen and stored at − 80 °C. Total protein was quantified using the Pierce BCA Protein Assay Kit. Cortical lysates were analyzed for IgG (1:3 dilution), with commercial ELISA kits (Immunology Consultants) with 2 minor modifications to the manufacturer’s protocol: sample incubation time was increased to 2 h at 37 °C, and secondary antibody was increased to 1 h at 37 °C. Standards were buffer matched with SDS lysis buffer. Plasma protein values were normalized to protein concentration.

### Statistical analysis

Data was analyzed in different ways (see Table 1 and Results overview). Briefly, for in vitro experiments, the effect of compound and LPS treatment was expressed by relative change (for a within-well normalization) and percent change or percent activity (as a within plate normalization). For statistical validation of the phenotypic screen, we used the interleaved plate layout as described in the NIH Assay guidance manual (Table 1). For compound response curves, our primary objective was to identify active concentrations. Thus, repeated measures one way analysis of variance (ANOVA) was used unless some values were missing where mixed-effects model (REML) was used instead. Once it was confirmed that there were differences between groups, we used Dunnett’s comparison of all concentrations of compounds versus either vehicle (compound phase) or LPS (LPS phase) for post-hoc analysis (*p* < 0.05) as recommended by prism (Version 10). For all in vitro data, *n* represents a different independent isolation of 16 mice per plate. For in vivo studies, data were analyzed by 2-way ANOVA followed by Tukey’s multiple comparisons tests. Outliers test was conducted by ROUT (1%) using GraphPad Prism Version 10.4.6. All data are represented as the mean ± standard error (SEM). All data point values, excluded data points with criteria, statistical analyses, n sizes, compound IDs, and compound information are available in Additional File 2 in the Supplemental Material (each tab corresponds to a figure).

**Table 1:** Definitions and statistical criteria utilized in this manuscript

Value	Equation/definition	Application
Vehicle/Vehicle	Vehicle control	There are two vehicles in this assay, one for the compounds (DMSO) and one for LPS (ECM).
Vehicle/LPS	LPS control	This has a vehicle for compound (DMSO) and LPS.
Capacitance	Instrument measured impedance at 64 kHz.	Indication of cell confluence. Values greater than 4nF indicate toxicity.
TEER	Instrument measured resistance at 1000 Hz	Permeability of the monolayer to ions. Lower values indicate higher permeability
Drug treatment phase: Relative change in TEER.	$\:\frac{{\text{24h post-compound}}_{treatment\:phase}}{\mathrm{p}{\mathrm{re-compound}}_{treatment\:phase}}$	Change in absolute values within a well after drug treatment. Lower values mean the drug disrupted TEER.
Drug treatment phase: Percent Change	$\:\left(\frac{\mathrm{R}\mathrm{e}\mathrm{l}\mathrm{a}\mathrm{t}\mathrm{i}\mathrm{v}\mathrm{e}\:\mathrm{c}\mathrm{h}\mathrm{a}\mathrm{n}\mathrm{g}\mathrm{e}\:\mathrm{c}\mathrm{o}\mathrm{m}\mathrm{p}\mathrm{o}\mathrm{u}\mathrm{n}\mathrm{d}}{\mathrm{R}\mathrm{e}\mathrm{l}\mathrm{a}\mathrm{t}\mathrm{i}\mathrm{v}\mathrm{e}\:\mathrm{c}\mathrm{h}\mathrm{a}\mathrm{n}\mathrm{g}\mathrm{e}\:\mathrm{V}\mathrm{e}\mathrm{h}\mathrm{i}\mathrm{c}\mathrm{l}\mathrm{e}}\right)x\:100$	Normalizes the relative change of a drug treated well to the vehicle within a plate (or n). Vehicles are 100% and lower values mean disruption. We used 85% as the cut-off value.
LPS phase: Relative change in TEER	$\:\frac{{\mathrm{18}\mathrm{h}\text{ post-LPS}}_{phase}}{{\mathrm{p}\mathrm{r}\mathrm{e}-LPS}_{phase}}$	Change in absolute values within a well after LPS treatment. Lower values indicate higher permeability.
LPS phase: Percent Activity	For LPS phase relative change values: ($\:\frac{\mathrm{(Vehicle}\,\,\mathrm{LPS}\mathrm{)-(}\mathrm{Vehicle}\mathrm{-}\mathrm{X}\mathrm{)}}{\mathrm{(}\mathrm{vehicle-LPS)}}$)x100	Within plate (n) normalization based on assay range. Vehicle is 100% and LPS is 0%. We used 50% as the cut-off value for an active compound.
Min	TEER, relative change and percent activity for 0.8 µg/ml LPS treated wells	For assay statistics this is the lowest value or highest permeability.
Max	TEER, relative change and percent activity for Vehicle treated wells	For assay statistics this is the highest value or lowest permeability.
Mid	TEER, relative change and percent activity for sildenafil (10 µM) treated wells	This value is used as the positive control. Also corresponds to the cut off for a positive hit in the screen.
Assay range	Max – min for either relative change of percent activity	For the relative change represents the difference between LPS and vehicle.
CV _min, mid, max_	$\left(\frac{\mathrm{SD}/ {\surd n}}{\mathrm{AVG}}\right)\times100$	This is a within plate calculation. For each 96 well plate the CV is calculated for the min, mid and max values. n represents the number of replicates to be used on the screen. All values should be below 20%.
Z’	$\:\frac{\mathrm{(}\text{AVG Max AVG Min-3(SD Max+SD Min))}{\surd}n}{{({\text {AVG Max}}{-} {\text{AVG Min}}})}$	This is a within plate value that is used to statistically validate the screen, taking into account the assay range and variation of the min and max values. Z’ > 0.4 is considered acceptable
SW (signal window)	$\:\frac{\mathrm{(}\text{AVG Max AVG Min-3(SD Max+SD Min))}{\surd}n}{\text{(SD Max/}{\surd}n)}$	Similar in concept to Z’. SW > 2 are considered acceptable.

## Results

### Overview of data presentation and assay calculations

Our goal was to determine whether *APOE4* brain endothelial cells can be used as a compound screen. To achieve that goal, we adapted our previously described *APOE4* brain endothelial cell isolation protocol and assay for permeability (LPS-induced lowering of TEER) according to suggestions in the NIH Assay guidance manual (see Table 1 for equations). Our overall strategy was to scale the isolation protocol, select conditions for the min (LPS), mid (LPS and positive control compound) and max (vehicle) signals, statistically validate the phenotypic assay, screen compounds, validate hits and test the top hits in vivo.

As this manuscript is a hybrid of assay development and screening results, data is plotted and analyzed in different ways. Therefore, although unconventional, we would like to provide a brief descriptive overview of the assay to help interpret the Figures and as a guide for others that may wish to conduct such a screen. After cells reach confluence there are two important phases that we call the compound treatment phase and LPS treatment phase.

The compound treatment phase is where compounds or vehicle control are added 24 h before the addition of LPS. This phase allows identification of compounds that are toxic or lower TEER values to exclude from analysis in the LPS phase. Data collected during method development, which for this study corresponds to Figs. 1, 2, 3 and 4, is frequently used to develop cut-off values for the main screen. For toxicity, typically > 10nF capacitance is considered toxic and we used > 4nF to increase sensitivity (e.g. Figure 3). To identify compounds that lower TEER, data were analyzed in two ways. The first is relative change in TEER, which corresponds to.

$\:\frac{{\text{24h post-compound}}_{treatment\:phase}}{\mathrm{p}{\mathrm{re-compound}}_{treatment\:phase}}$ and accounts for the fact that individual wells may have slightly different starting absolute values. To set cut-off values, the relative change was converted to percentage activity within each plate with the equation $\:\left(\frac{\mathrm{R}\mathrm{e}\mathrm{l}\mathrm{a}\mathrm{t}\mathrm{i}\mathrm{v}\mathrm{e}\:\mathrm{c}\mathrm{h}\mathrm{a}\mathrm{n}\mathrm{g}\mathrm{e}\:\mathrm{c}\mathrm{o}\mathrm{m}\mathrm{p}\mathrm{o}\mathrm{u}\mathrm{n}\mathrm{d}}{\mathrm{R}\mathrm{e}\mathrm{l}\mathrm{a}\mathrm{t}\mathrm{i}\mathrm{v}\mathrm{e}\:\mathrm{c}\mathrm{h}\mathrm{a}\mathrm{n}\mathrm{g}\mathrm{e}\:\mathrm{V}\mathrm{e}\mathrm{h}\mathrm{i}\mathrm{c}\mathrm{l}\mathrm{e}}\right)x\:100$, which considers potential cross plate variation (discussed below). For the percentage activity, vehicle represent 100% and we set the cut-off value at 85% i.e. 15% lowering of TEER.

In the LPS phase, LPS or vehicle is spiked into the well. The relative change corresponds to.

$\:\frac{{\mathrm{18}\mathrm{h}\,\,\mathrm{post-LPS}}_{phase}}{{\mathrm{p}\mathrm{r}\mathrm{e}-LPS}_{phase}}$, and accounts for within well variability. To standardize across plates, as for the compound treatment phase, we calculated percentage activity: ($\:\frac{\mathrm{(Vehicle}\,\,\mathrm{LPS}\mathrm{)-(}\mathrm{Vehicle}\mathrm{-}\mathrm{X}\mathrm{)}}{\mathrm{(}\mathrm{vehicle-LPS)}}$)x100. Thus, percentage activity is within plate analysis of compound activity, where LPS represents 0% and vehicle is 100% for each plate/n. We set 50% as the cut-off value for a positive hit i.e. 50% higher than LPS.

Percentage activity is the typical parameters utilized for assigning compounds as hits as per the NIH Assay Guidance. We want to echo the utility of percentage activity and highlight the advantage of its use for this phenotypic screen. Candidly, in our scaled brain endothelial cell isolation we have found some variation in how the vehicles behave for each n/plate in the treat and LPS phases. In the compound treatment phase, the relative change for a vehicle was typically around 1 but could vary between 0.9 and 1.2. In the LPS phase the vehicle had a relative change that varied from 0.8 to 1. Although the relative change of the vehicle could vary across plates, compound and LPS responses were proportionally consistent. For example, in the LPS phase even if the relative change of the vehicle was < 1 (e.g. 0.8), LPS caused a more pronounced drop (e.g. 0.52). Importantly, when assessed by percentage activity the responses to a compound or LPS are similar across plates. Therefore, we have plotted the data in two ways throughout the manuscript, as relative change and percent activity.

### Scaling the brain endothelial cell isolation

Our previous brain isolation protocol was designed to conduct experiments at passage zero to limit phenotypic changes (e.g., protein expression, functions, loss of specialization) that occur with cell passage, cell freezing and longer culture time. That protocol was developed for mechanistic studies, where one mouse corresponds to an *n* (one cortex produces 8 wells of a 96 well plate). As our goal was to screen compounds on 2–4 × 96 well plates per week, we first had to amend the isolation protocol to increase scale (detailed in Methods). The major differences compared to single brain isolations were that cortices from 16 to 18 mice were pooled per 96 well plate (prediction was 12 based on [18], digest buffer volumes were lower per brain and the first wash step after isolation was conducted 16 h instead of 6 h later. We made the changes as we found that the isolation did not scale proportionally, and the cells were more sensitive to detaching after plating in the scaled version.

In our modified isolation protocol, cell characteristics were slightly different than for single brain isolations [18]. To measure brain endothelial cell confluence and permeability we utilized electrical cell-substrate impedance sensing on the ECIS^®^ ZΩ (Applied Biosystems) as it uses a 96-well plate format, which was critical for compound screening. Brain endothelial cells are grown on electrodes through which a small alternating current is passed to enable quantification of different parameters. Impedance at higher frequencies (64 kHz) is most affected by cell coverage (i.e., confluence, surrogate toxicity marker), whereas at lower frequencies (1000 Hz) more of the current passes between and through the cells and measures permeability (TEER). In our previous studies with single brain isolations, cells reached confluence 48 h after plating and remained stable with experiments complete after 7 days. In our scaled version it takes longer for cells to reach confluence (96 h); after reaching a plateau there is a slow decline until steady state is reached (~ 140 h) and the cells are sensitive to full media changes but recover and remain stable (Fig. 1A). Although we tried several adjustments (and are continuing to try), we were unable to get pooled brain isolations to grow the same as single brain isolations. Thus, the finalized timeline was isolation on day 0, puromycin added on day 1 (to kill non-brain endothelial cells), puromycin removed on day 3, media changed on day 6 (+/- compound, treatment phase), LPS spiked on day 7 (LPS phase) and experiments completed on day 8.

### Selecting a concentration of LPS for the min signal: 0.8 µg/ml

We focused on selecting a concentration of LPS that produced sustained permeability disruption but was non-toxic with the modified isolation protocol. One of our future studies is to understand how brain endothelial cells respond to different aging and neurodegeneration relevant stressors, however, so far, we have characterized LPS. Although there are limitations (see Discussion), LPS is a general inflammatory stressor that may have some signaling relevance for neurodegeneration [37–39] and we have found that *APOE4* brain endothelial cells are particularly vulnerable to LPS-induced TEER disruption [18]. Therefore, as a proof-of-concept study we used LPS-induced TEER disruption assay as our phenotypic screen.

We evaluated the impact of LPS over a range of 0.001–22 µg/ml on capacitance (toxicity) and TEER over 24 h. As we found previously, LPS does not affect capacitance at concentrations up to 22 µg/ml, (Fig. 1B-C) in *APOE4* brain endothelial cells. Key for evaluating compound activity is a comparison of pre- and post- LPS spike TEER, which we calculated in two ways. The first was raw resistance values (Fig. 1D-E) and the second relative change, that is post-LPS/pre-LPS (Fig. 1F-G) to account for well variability in starting TEER values. The relative change produced more consistent data that was similar to our LPS results with single brain isolations. When evaluated by relative change, LPS caused a reduction in TEER over 6 h that either recovers and is higher than vehicle, or recovers and is similar to vehicle or remains low (Fig. 1F). Thus, after 18–24 h treatment there are three clusters of LPS concentrations compared to vehicle (Fig. 1G): higher (0.001 µg/ml), no effect (0.003–0.09 µg/ml) and lower (0.3–22 µg/ml). The higher TEER with low concentrations of LPS is consistent with data from other groups [40]. As we wanted to use an LPS concentration that induced a sustained lowering of TEER we selected 0.8 µg/ml at the 18 h time point and relative change as the readout for subsequent steps of screen development.

### Identifying a positive control for the mid signal: 10 µM sildenafil

For statistical evaluation of a phenotypic screen a positive control compound is required, which ideally has ~ 50% activity (mid signal). We previously found that the Rev-Erb agonist SR9009 (Stenabolic) could partially prevent LPS-induced TEER disruption of *APOE4* brain endothelial cells. Therefore, we evaluated whether SR9009 (0–10 µM) could serve as our positive control (Fig. 2A). In the compound treatment phase, we found that SR9009 was non-toxic (Fig. 2B) but lowered TEER (Fig. 2C-D) by greater than 15% at higher concentrations (2.5–10 µM). In the LPS phase (Fig. 2E-F), at lower concentrations SR9009 partially prevented TEER disruption, however the activity was not at 50%. Overall, SR9009 had a very narrow therapeutic window, and we also encountered difficulties in maintaining solubility which made it sub-optimal as a positive control.

We next focused on identifying a different positive control and tested phosphodiesterase (PDE) inhibitors. cAMP and cGMP initiate signaling cascades considered protective for vascular function and PDEs degrade cAMP (PDE3), cGMP (PDE5, PDE9) or both (PDE2). PDE inhibitors were developed for peripheral cardiovascular disorders [41] and could protect brain endothelial cells [42–45]. We performed a mini screen of inhibitors for PDE2 (EHNA, BAY60-7550), PDE3 (cilostazol, milrinone, anagrelide, vesnarinone), PDE5 (Dipyridamole, vardenafil, sildenafil, zaprinast) and PDE9 (BAY73-6691) at three different concentrations (1,10 and 100 µM, Fig. 3A-F). In the compound treatment phase only a few concentrations of select compounds were toxic or lowered TEER (Figs. 3B-D and 100 µM anagrelide, 10 and 100 µM dipyridamole, 100 µM BAY60-7550, EHNA and sildenafil) and were removed from subsequent analysis. In the LPS phase, vardenafil, sidenafil, vesnarinone, cilostazol and EHNA were active at limiting LPS-induced lowering of TEER (Fig. 3E-F). In expanded concentration-response curves (Fig. 3G-L), in the LPS phase vardenafil (*≥* 0.39 µM), sildenafil (*≥* 1.56 µM), EHNA/cilostazol (*≥* 12.5 µM) and vesnarinone (*≥* 50 µM) mitigated TEER disruption when assessed by relative change. When assessed by percent activity vardenafil and sildenafil (≥ 3.125 µM), EHNA (≥ 25 µM) and cilostazol (≥ 50 µM), met the 50% cut-off criteria. These data have interesting mechanistic implications as they imply that the cAMP and /or cGMP may be protective for *APOE4* brain endothelial cells (see Discussion).

We selected sildenafil as our positive control based on several considerations. First, sildenafil (Viagra) is becoming widely used and therefore has clinical implications. Second, sildenafil was active at lower concentrations than EHNA, cilostazol and vesnarinone. Third, concentration response curves for sildenafil were more typical than for vardenafil. Visually, vardenafil was very active at low concentrations, plateaued sharply and then at higher concentration jumped in activity or was toxic. Mechanistically, vardenafil may warrant more detailed evaluation, however for our screen we wanted to avoid more atypical compounds and so we selected sildenafil.

To determine the window of detecting protective effects of sildenafil we performed a dose-response curve of LPS, +/- 1 µM or 10 µM sildenafil (Fig. 3M-Q). We found that 0.8 µg/ml LPS with 10 µM sildenafil was the most sensitive, as the difference in relative change was the highest compared to other concentrations of LPS. In addition, at 0.8 µg/ml LPS the assay appeared most sensitive, as 1 µM sildenafil also showed protective effects. This data further supports the selection of 0.8 µg/ml LPS as it produced a strong, consistent decrease in TEER without toxicity, providing a sufficient assay window for identifying protective compounds.

### Phenotypic screen statistical validation

We next evaluated whether our phenotypic assay met the reproducibility acceptance criteria for a statistically validated assay. In the previous experiments, we selected conditions for min (0.8 µg/ml vehicle/LPS), mid (10 µM sildenafil/LPS) and max (vehicle/vehicle). Acceptance criteria are based on reproducibility/variance of the mid, min and max values within plates, across plates and across days. The specific criteria are as follows: Outliers < 2%; %CV for max, mid, min ≤ 20%; Z’ ≥ 0.4; No edge or drift across the plates; Normalized average mid-signal should not have > 2-fold shift within or across days (see Table 1 for list of calculations).

We used the interleaved format of two plates on two different days (Fig. 4A-B) to statistically evaluate our screen using both the relative change (Table 2A) and our focus percent activity (Fig. 4C, Table 2B). There were no outliers, however occasionally wells failed to give readings that were related to manufacturing issues rather than experimental issues and visually there were no or edge and drift effects (Fig. 4B). For relative activity, LPS reduced TEER values by ~ 35% and by 22% with sildenafil treatment and so the assay range is 0.35 (~ 1.6 max/min) (Data from Fig. 4C was used in calculations for Table 2A-B). When converted to percent activity sildenafil at 10 µM was ~ 40–50%. For both relative activity and percent activity for 3 repetitions, the CVs of max, mid and min were below 20%, Z’ scores were all greater than 0.4 (0.44–0.76) and the normalized average mid-signal did not have > 2-fold shift within or across days (Table 2A-B). Thus, our assay met the guidelines for a statistically validated assay.

**Table 2:** Statistical screen validation A for % Activity and B for relative change. Assay type: activation; number of replicates per concentration to be used in production assay: 4; typical value for slope of dose-response curve: 1. Acceptance criteria for the validated assay (NIH assay guidance manual) were as follows: all max (HI) signal CV’s < 20%, all unnormalized mid signal (mid %) SD’s < 20, all SW’s > 2, all Z’ Factors > 0.4, all within-day fold shifts < 2, all average (between-) day fold shifts < 2

A.	High			Mid		Low					
	Mean	SD	CV	Mean	CV	Mean	SD	CV			
Day 1, Plate 1	100	17.97	10.38	27.60	17.94	0.00	14.35	NA			
Day 1, Plate 2	100	13.18	7.61	33.93	19.96	0.00	7.64	NA			
Day 2, Plate 1	100	9.44	5.45	42.57	9.10	0.00	4.55	NA			
Day 2, Plate 2	100	14.55	8.40	51.27	11.87	0.00	7.56	NA			
	SW	Z'	%Spec	Max/Min	Day Ave.	Mid% diff within days	EC50/IC50/Ki	Meets criterion?	Mid % between days	Meets criterion?	
Day 1, Plate 1	4.24	0.44	100	-3.56E+15	30.76	-6.33	1.35	Yes	1.98	Yes	
Day 1, Plate 2	8.40	0.64	100	1.04E+16							
Day 2, Plate 1	13.90	0.76	100	-3.02E+15	46.86	-8.58	1.41	Yes			
Day 2, Plate 2	7.35	0.62	100	2.02E+15							
Validation Checklist											
Intra-Plate Tests					Meets Criterion?						
All max (HI) signal CV's < 20%					Yes						
All mid signal (unnormalized) CV's < 20%					Yes						
All normalized mid signal (mid %) SD's < 20					Yes						
All min (LO) SD's < Min(max (HI) SD, mid SD)					No						
All SW's >2					Yes						
All Z' Factors > 0.4 (and < 1 ; must pass one of 6 or 7)					Yes						
Inter-Plate Tests											
All within-day fold shifts < 2					Yes						
All Average (between-)Day fold shifts < 2					Yes						

B.	High			Mid			Low				
	Mean	SD	CV	Mean	SD	CV	Mean	SD	CV		
Day 1, Plate 1	0.99	0.07	3.91	0.72	0.03	2.56	0.62	0.05	5.00		
Day 1, Plate 2	0.96	0.05	2.93	0.71	0.04	3.50	0.59	0.03	2.77		
Day 2, Plate 1	1.04	0.04	1.98	0.83	0.03	1.77	0.66	0.02	1.49		
Day 2, Plate 2	0.98	0.04	2.49	0.84	0.03	2.11	0.69	0.02	1.84		
	SW	Z'	%Spec	Max/Min	Mid% data	Day Ave.	Mid% diff within days	EC50/IC50/Ki	Meets criterion?	Mid % between days	Meets criterion?
Day 1, Plate 1	4.24	0.44	38	1.60E+00	27.60	30.76	-6.33	1.35	Yes	1.98	Yes
Day 1, Plate 2	8.40	0.64	39	1.63E+00	33.93						
Day 2, Plate 1	13.90	0.76	36	1.57E+00	42.57	46.86	-8.58	1.41	Yes		
Day 2, Plate 2	7.35	0.62	30	1.42E+00	51.15						
Validation Checklist											
Intra-Plate Tests							Meets Criterion?				
All max (HI) signal CV's < 20%							Yes				
All mid signal (unnormalized) CV's < 20%							Yes				
All normalized mid signal (mid %) SD's < 20							Yes				
All min (LO) SD's < Min(max (HI) SD, mid SD)											
All SW's >2							Yes				
All Z' Factors > 0.4 (and < 1 ; must pass one of 6 or 7)							Yes				
Inter-Plate Tests											
All within-day fold shifts < 2							Yes				
All Average (between-)Day fold shifts < 2							Yes				

### Phenotypic screen

Our next goal was to conduct the phenotypic compound screen. Our finalized workflow was: day 0 is isolation, day 1 add puromycin, day 3 media change, day 6 compound treatment phase and day 7 LPS phase (Fig. 4D). On each 96 well plate there were three repetitions of each compound along with assay control wells for the min, mid and max signals (Fig. 4E). In the compound treatment phase, we set criteria where compounds that were toxic (capacitance of *≥* 4nF) or lowered TEER by 15% or greater were flagged for exclusion (Fig. 4F). In the LPS phase, percentage activity of *≥* 50% is considered a hit. For screening, we selected a subset of ~ 900 molecules from the TargetMol Bioactive Library at 1µM (see Additional File 2, Tab Compound ID). The compounds were inhibitors for a broad range of signaling molecules, which were entirely compound-like scaffolds, with well-known structure-activity relationships and many are FDA approved. We selected the library for a broad range of targets with multiple compounds per target in many cases, as our overall objective was assessing the potential of *APOE4* brain endothelial cells to serve as a screen.

### Compound treatment phase: mTOR inhibitors disrupt *APOE4* brain endothelial cells

In the treatment phase 11 compounds were toxic to *APOE4* brain endothelial cells (Fig. 5A). Seven of the compounds targeted kinases that are involved in gene transcription and apoptosis including CDK, TOPK, peptidyl transferase and Bcr-Abl and so toxicity is somewhat expected. One toxic compound inhibited pyruvate kinase, which is the major enzyme of glycolysis and three of the compounds inhibited PI3K/mTOR which induces multiple effects on cells including regulation of metabolism.

After excluding toxic molecules, we unexpectedly found five compounds that substantially increased TEER/lowered permeability by 20% or greater (Fig. 5B-C). Two of the compounds are MEK1/2 inhibitors, which is downstream of several receptor tyrosine kinases. This data may imply that baseline MEK activation increases permeability in *APOE4* brain endothelial cells and could serve the basis of additional mechanistic studies. In the opposite direction, 34 compounds lowered TEER/increased permeability by ~ 15% or greater. Of those 34, 19 are inhibitors of mTOR or PI3K (Fig. 5B-C). mTOR inhibitors were particularly disruptive as ridaforolimus, VS-5584, torin 1, zotarolimus, everolimus and rapamycin all lowered TEER by ~ 50%. Consistent with that result we found that rapamycin treatment of *APOE4* brain endothelial cells resulted in lower levels of phosphorylated 4EBP (compared to total), which is a downstream target of mTOR. That result suggests that the effects of rapamycin on baseline TEER disruption are mTOR mediated (Additional File 4, Supplementary Fig. 1A-C). We also tested the impact of rapamycin treatment on LPS-induced TEER disruption. We found that rapamycin did not impact LPS-induced TEER disruption when administered before, at the same time, or after LPS (Supplementary Fig. 1D-R). Thus, although mTOR inhibition disrupted baseline TEER, it did not exacerbate or mitigate LPS effects on permeability. In addition, we evaluated whether the impact of mTOR inhibition on TEER was *APOE4* specific, by treating *APOE3* and *APOE4* brain endothelial cells with rapamycin (Supplementary Fig. 1S-U). We found that rapamycin resulted in lower TEER values in both *APOE* genotypes to a similar extent. Overall, our data suggests that acute mTOR inhibition disrupts TEER in brain endothelial cells in vitro.

As mTOR inhibition increased *APOE4* brain endothelial cell permeability we tested the concept that mTOR activation may be beneficial for preventing LPS-induced TEER disruption using the agonists NV-5138, L-leucine, 3BDO and MHY1485. After excluding concentrations that were toxic, only higher doses of NV-5138 (100–200 µM) and 3BDO (100 µM) showed some protection against LPS-induced TEER disruption. Although those data were somewhat promising, high concentration of mTOR agonists were needed for activity, and the dose response curves were somewhat varied and atypical (Fig. 5E-I). Therefore, we decided not to test mTOR agonists in vivo. Nonetheless our data supports that inhibiting mTOR is detrimental for *APOE4* brain endothelial cell function and that activating mTOR to some extent can protect against LPS-induced TEER disruption in vitro.

### LPS phase: SGI-7079, Vorinostat and CCT196969

We next moved onto evaluation of compounds that were non-toxic and did not disrupt TEER in the LPS phase (Fig. 6A-D). In this phase, the activity of sildenafil was 50% which was our cut off for positive hits. We found 33 compounds that met the criteria that could be categorized into different classes. 15 of the compounds were inhibitors of tyrosine kinase receptors including AXL, VEGFR, EGFR, c-Met, ALK and TGFβR. Those data are consistent with reports that have linked independent effects growth factor receptors to different functions of LPS (see discussion). Therefore, LPS may activate multiple different types of tyrosine kinase receptors. Of the remaining compounds, they could be grouped into those that target the cell cycle/apoptosis (5 compounds), MAPK (4 compounds), JAK (2 compounds), CaMK (2 compounds) and other pathways (Raf, AKT, PKD, PI4K), consistent with the idea that growth factor receptors and LPS can activate pleiotropic signaling cascades.

As our goal was transition hits to in vivo testing, we conducted concentration-response curve validation (0.01-10 µM) with five of the most active compounds; SGI-7079 (AXL inhibitor), vorinostat (HDAC inhibitor), semaxinib (VEGFR inhibitor), CCT196969 (raf inhibitor) and tofacitinib (Jak inhibitor) (Fig. 6E-I). In the compound treatment phase, a few concentrations were toxic or lowered TEER values (SGI-7079; ≥2.5 µM, CCT196969 at 10 µM). Interestingly, vorinostat (5 µM) and CCT196969 (2.5 µM and 5 µM) increased TEER values by ~ 25%, suggesting that HDAC and AXL play a role in regulating baseline permeability.

In the LPS phase, tofacitinib and semaxinib were active at lowest dose tested (0.01 µM ~ 60% activity) but activity did not increase with higher doses and in some cases appeared lower, which indicates a saturation of activity at low concentrations. These data support that JAK and VEGFR signaling is involved in LPS-induced TEER disruption to some extent. SGI-7079 (≥ 0.31 µM, vorinostat (≥ 0.31 µM) and CCT196969 (≥ 1.25 µM treatment were also protective. In terms of percentage activity, those three compounds surprisingly were greater than 100%, which was particularly pronounced for vorinostat (200% at 10 µM) and CCT196969 (152% at 5 µM) which indicates a potentiation of recovery.

Collectively, these data support that our screen could identify compounds that prevent LPS-induced TEER disruption to *APOE4* brain endothelial cells.

### Treatment with top hits or tadalafil prevent LPS-induced IgG brain permeability in mice

Our final goal was to determine whether compounds identified in the screening phase were active in vivo (Fig. 7A). As described above the compounds we selected from the screen were SGI-7079, CCT196969 and vorinostat. Consistent with the proposed mechanism of action of each compound, we found that vorinostat increased levels of acetyl-H3/total H3 levels after LPS treatment (Additional File 4, Supplementary Fig. 2). In addition, we wanted to include a PDE5 inhibitor as a positive control, similar to our in vitro approach. As we were about to conduct our in vivo study it was brought to our attention tadalafil (Cialis) is increasingly used over sildenafil for erectile dysfunction [46–48]. We therefore tested the activity of tadalafil in vitro and found it raised baseline TEER in the drug phase and therefore partially mitigated LPS-induced disruption (Additional File 4, Supplementary Fig. 3). Although tadalafil appears less active that sildenafil in vitro, a major advantage of tadalafil over other PDE inhibitors for non-erectile vascular dysfunction in vivo, particularly in context of neurodegenerative disorders, is a favorable pharmacokinetic profile [49]. By design, the majority of PDE inhibitors are rapidly cleared from the plasma (2–4 h complete removal), whereas tadalafil is cleared at much lower rates and plasma levels remain 24 h after treatment. Therefore, we performed a pilot where we treated (i.p.) female *APOE4* mice with either vehicle control, tadalafil (20 mg/kg) [33], CCT196969 (20 mg/kg) [34], SGI-7079 (50 mg/kg) [35] or vorinostat (50 mg/kg) [36], followed 24 h later with either PBS or lipopolysaccharide (LPS; 1 mg/kg, i.p.) to induce brain endothelial cell permeability (Fig. 7B). We used female mice because we have found that the combination of *APOE4* and female sex increases susceptibility to age-related brain endothelial cell dysfunction [17]. The LPS dose was selected to increase permeability without inducing sickness behavior [50] and is based on a previous experiment in our lab using wild type mice (data not shown). The doses of each compound were selected based on published data from other groups demonstrating positive activity in vivo (referenced next to each compound above).

After LPS treatment, all mice showed a body weight loss of 10% compared to vehicle treated mice indicating an effective LPS-induced general inflammatory response **(**Fig. 7C**)**. One way to measure brain endothelial cell permeability, is to assess levels of plasma proteins in the brain that do not readily cross unless the cells are dysfunctional, and we measured IgG levels [51–53]. In the cortex, levels of IgG were ≈ 40% higher in LPS treatment mice compared to the vehicle treated group. Importantly, cortical IgG levels were lower in mice pretreated with all 4 compounds followed by LPS (Fig. 7D**)** compared to the vehicle/LPS treatment group. These data support that compounds identified with our in vitro approach are active in vivo at preventing acute LPS-induced increases in brain endothelial cell permeability.

## Discussion

Despite their critical role in cerebrovascular function, brain endothelial cells are often overlooked in compound discovery efforts. As proof-of-concept, one of our goals was to evaluate the feasibility of using primary brain endothelial cells that express *APOE4* as a compound screen. Overall, we propose that the assay is suited for phenotypic screening as the isolation was scalable and the assay (LPS-induced TEER disruption) met statistical criteria. In addition, we were able to identify and validate hits with a relatively stringent cut-off criteria, whose targets are biologically logical given what is known about LPS (see next paragraph). For any groups, including our own, that wish to conduct this type of research there are some important things to consider. First is the feasibility and effort. We provided in Additional file 3 a rough estimate of reagents and time needed. In general, the development stage took us ~ 1 year, given the issues we had with scaling the isolation, identifying a positive control and developing the workflow (e.g. ordering reagents, aging mice ready for breeding trios). Once we were set up, screening 900 compounds took ~ 15 weeks of continual experiments. Critical steps were setting up sufficient breeding trios (we used ~ 30) to enable 2–4 plates of screening every week. Some researchers may be tempted to freeze or pass cells to reduce trio number or to aid in timing the experiments. We strongly advise against doing that, as one of the main advantages of the screen is the use of primary cells without sub-culture, freezing and passing which alters the brain endothelial cell phenotype. To aid in continuous screening, we purchased four systems to measure TEER in different plates simultaneously and as a backup in the event a computer or machine goes down. In addition, it is important to order the specialized TEER plates in a staggered manner, as they are only guaranteed stable for a few months. A key step is ordering and stocking key reagents. As examples, for screening we used 80 papain vials, 40 DNAse vials, 880 ml of BSA and 320 × 96 boxes of filter tips. Second is that plotting the data as relative change and percentage activity in both the drug treatment and LPS phases was extremely helpful for interpreting and plotting data. Third is the drug treatment phase. We used 24 h, however this could be longer or shorter depending on when the desired stressor is to be added. That phase was critical for enabling the removal of compounds that are toxic and non-toxic but disrupt TEER. This stage also can help identify pathways that regulate baseline permeability in the cell line of choice. Fourth is initial library selection. We used a library of compounds with known targets that contained multiple compounds that target the same target or pathway, which allows confidence in assigning hits. Non-targeted libraries could then be used. Fifth is the potential applications of the screen. This screen could be used to identify protective compounds for diseases associated with higher brain endothelial cell permeability for which there are disease models. For example, disease relevant stressors can be added to any source of brain endothelial cells and/or brain endothelial cells could be isolated from models of genetic disease. Specifically, the model could be adapted to include oxygen glucose deprivation to mimic stroke, Aβ to mimic cerebral amyloid angiopathy and brain endothelial cells isolated from genetic mice with NOTCH3 manipulations could be used to mimic small vessel disease. In many ways the reductionist approach is attractive for focusing on brain endothelial cell specific pathways.

Although our main goal was a screening feasibility assessment, it is important to discuss potential mechanistic and therapeutic implications of our data. In the compound treatment phase, we found that mTOR inhibitors caused TEER disruption. mTOR is a kinase with multiple functions including metabolism, cell growth/survival and has been linked to cancer and neurodegenerative disorders (reviewed in [54]). In general, data on whether mTOR inhibition is beneficial or detrimental for a disease is conflicted since mTOR is expressed by all cell types in the body and has multiple functions. In terms of age-related disorders, most studies have used rapamycin to evaluate the role of mTOR in vivo. Some studies have reported a positive effect on behavior and Aβ levels in human APP/Aβ mouse models [55]. However, a recent paper demonstrated that enhancing mTOR signaling (via S1P analogues) can mitigate behavior dysfunction induced by Aβ injections [56] and there are contrasting results on whether rapamycin is ineffective or beneficial at modulating Aβ levels (reviewed in [57]). Therefore, in the context of behavior and pathology there is still a lack of consensus on whether mTOR inhibition is beneficial in Aβ models.

Specific for cerebrovascular function, rapamycin treatment of human APP/Aβ mouse models has been found to protect against blood-brain barrier leakiness [58] and neurovascular coupling deficits [59]. In addition, in *APOE4*-Aβ models rapamycin treatment resulted in improved cerebral blood flow and other metrics of brain function [60, 61]. That data seemingly contrasts with our data that mTOR inhibition increases permeability in *APOE3* and *APOE4* brain endothelial cells. There are several potential explanations. One is that the impact of mTOR inhibition on other cells, including neurons and glia, is beneficial for brain endothelial cell function and behavioral in context of aging and neurodegenerative models. For example, cytokines and chemokines produced in the brain can disrupt brain endothelial cell function and could be prevented by mTOR inhibition. If that idea is correct, then it highlights the potential limitations of the in vitro model. A second explanation is that the role of mTOR on brain endothelial cell functions such as permeability or vasodilation responses are treatment duration dependent. Indeed, although it has been proposed that mTOR inhibition will be beneficial for endothelial cell responses (reviewed in [62]), there is data supporting an opposing view. For example, in the acute phase of epilepsy model, rapamycin has been found to exacerbate brain endothelial cell leakiness and also increases susceptibility to dysfunction, however the effect may be brain region and timing dependent [63, 64]. In addition, drug eluting stents, used to treat coronary artery disease, are associated with delayed healing and impaired vasodilation responses in some patients. The stents that contain rapamycin and mTOR inhibition have been shown to disrupt vasodilation responses [65, 66], likely due to eNOS suppression and uncoupling. Further, mTOR activity may participate in recovery after ischemia and contribute to the protective effects of ischemic post conditioning [67–70] and inhibiting mTOR is associated with cardiovascular dysfunction [71]. The conditions where mTOR inhibition is detrimental for vascular function may be related to a shorter-term effect, that could be analogous to our in vitro system. A third possibility is that mTOR inhibition can be beneficial or detrimental depending on the context of brain endothelial cell signaling. In general, there is a high possibility that too low or too high mTOR signaling is detrimental for brain endothelial cell function, which is disease specific. Our data implies that in baseline conditions mTOR plays an important role in maintaining the barrier function of brain endothelial cells, which raises the question of mechanism. mTOR is linked to several functions including metabolism, protein and lipid synthesis and autophagy any of which could impact brain endothelial cell permeability. Speculatively, we believe that certain aspects of metabolism could play a role in how mTOR regulated brain endothelial cell function. Although not without controversy, there is a concept that glycolysis in endothelial cells is important for energy production [72–74], perhaps to maintain the supply of oxygen into the tissue. mTOR is often referred to as a master regulator of metabolism and promotes glycolysis. Therefore, at baseline, brain endothelial cells may be particularly susceptible to glycolytic disruption due to mTOR inhibition. In addition, given that vorinostat (HDAC inhibitor) and PDE5 inhibitors raised baseline TEER in this current study, perhaps inhibiting mTOR disrupts either HDAC signaling and/or the nitric oxide-cGMP pathway. Further research is needed to uncover the complex cell-type and context-dependent specific functions of mTOR.

In the LPS phase, we found compounds that protected against TEER disruption that fell into different classes. One class were inhibitors of growth factor receptors, including for EGFR, VEGFR, AXL, ALK, c-Met, and TGFβR. These results are consistent with reports from several independent groups that have linked LPS and/or sepsis to growth factor signaling. For example, LPS has been found to transactivate the EGFR [75] and blocking the EGFR with erlotinib is protective against LPS-induced toxicity in vivo [76–79]. In terms of other growth factors, LPS has been shown to upregulate VEGF expression [80, 81], enhance TGF-β1 signaling [82] and increase shedding of AXL [83]. Further LPS-induced cytokine/chemokine production is partially lowered by silencing/inhibiting AXL and ALK [84] and LPS can increase epithelial cell permeability through c-Met activation [85]. LPS likely impacts those growth factor receptors through pleiotropic mechanisms of action, such as transactivation, induction of growth factor production, activation by cytokines and chemokines secreted in the media and/or intracellular signaling interactions. In terms of downstream cascades, we found that inhibiting proteins that have different functions/signaling cascades could partially mitigate LPS-induced TEER disruption. They included those involved in the cell cycle, MAPK, JAK, calcium signaling and with a less stringent cut-off criteria, modulators of the cytoskeleton. As for the growth factor receptors, all these pathways have been found to be associated with LPS-induced effects in vitro (e.g. cell cycle [86, 87], MAPK/JAKSTAT [88–90], CAMK2 [91], FAK [84]), which is logical given that TLR4 and growth factor receptors signal through many signaling pathways all of which can impact brain endothelial cell permeability. Overall, it was reassuring that through using a broad range of inhibitors in our assay we were able to corroborate the independent findings of how LPS can signal in vitro.

Based on in vitro activity, we tested tadalafil (PDE5 inhibitor), vorinostat (HDAC inhibitor), CCT196969 (raf inhibitor) and SGI-7079 (AXL inhibitor) in vivo and found they were protective. Thus, those compounds may have relevance in the context of neurodegenerative disorders. Tadalafil is important for nitric oxide (NO)/soluble guanylate cyclase (sGC)/cGMP signaling. NO activates sGC to increase cGMP production which activates protein kinase G (PKG1 and 2) and some ion channels. cGMP is degraded by PDE, including PDE5. cGMP is considered beneficial for vascular and metabolic function and disrupted signaling contributes to cardiovascular and metabolic diseases. Thus, PDE5 inhibitors are used clinically for treatment of erectile dysfunction, pulmonary hypertension, heart failure and Reynaud’s disease (reviewed in [92–96]). Previous studies have also found that PDE5 inhibitors can protect against different types of LPS-induced inflammatory responses in vitro and in vivo [97–101]. There is also evidence that PDE5 inhibitors could be beneficial for neurodegeneration [41, 92, 96, 102–105]. For example, PDE5 inhibitor use has recently been associated with lower Alzheimer’s disease risk in epidemiological/data observation studies [103, 106–109] protection in small vessel disease [110] and other conditions of cognitive dysfunction [111]. In addition, several in vitro and in vivo studies have reported positive effects of PDE5 inhibition in models of Alzheimer’s disease [112–115]. However, contrary studies contest the effects of PDE5 inhibitors in reducing AD risk [116] or small vessel disease [117]. Thus, there is debate on the merits of PDE5 inhibition in AD [118, 119]. Our data would suggest that PDE5 inhibitors may be useful for targeting patients for which blood brain barrier dysfunction contributes to neural dysfunction and could include *APOE4* carriers. The caveat is that the mechanism through which LPS induces brain endothelial cell dysfunction and that found in neurodegenerative disorders may be different.

Vorinostat was developed for cancer therapy [120, 121] and is used to treat cutaneous T-cell lymphoma [122]. As an HDAC inhibitor, vorinostat regulates different aspects of cell division, differentiation and apoptosis [123–125]. In addition to cell division/cell cycle, increasing evidence suggests that HDAC regulates several functions including inflammation and synaptic function. Indeed, previous studies have shown beneficial effects of HDAC inhibitors in LPS/sepsis [126–129]. There is increasing evidence that HDAC inhibitors, including vorinostat may be effective in neurodegenerative disorders [130]. In the case of Alzheimer’s disease (reviewed in [130, 131] vorinostat can improve memory and neuron function in Aβ models [132, 133]. In contrast, in a separate study, vorinostat did not improve brain function, which may relate to the formulation [134]. Interestingly as HDAC inhibition may cause side effects with long-term use in a disease like AD, low dose tadalafil with low dose vorinostat have been tested in combination in Aβ models and improved behavior [135]. As for PDEs, it may be important to determine the extent that HDAC contributes to brain endothelial cell dysfunction in neurodegenerative disorders [136].

Relatively less is known on the role of CCT196969/raf and SGI-7079/AXL inhibitor in neurodegeneration. The ras/raf/MAPK pathway is well known in cancer research, with mutations or aberrant activation promoting cell division [137]. There is also a tight coupling of growth factor receptors, including EGF, to ras/raf signaling further strengthening the importance of the pathway in cancer research [137]. Consistent with our data, in other cell lines, LPS has also been shown to signal through raf1 [138–141]. In terms of neurodegeneration, higher raf1 levels/activation have been identified in Alzheimer’s disease patients [142, 143]. For Axl there is less literature (reviewed in [144]), but includes that AXL may be involved in microglial Aβ uptake, regulation of apoE levels and stress-induced endothelial cell dysfunction [145–147]. However, whether changes in raf1 and AXL are an adaptive response, detrimental, cause or consequence of neurodegenerative changes is unclear.

### Limitations

There are limitations in every approach to science, which are important to acknowledge to manage expectations and provide context for data. One limitation is that we used a reductionist approach to focus solely on brain endothelial cells. If a research question is more focused on the interaction between brain endothelial cells and cells that support neurovascular function (e.g. astrocytes, pericytes, microglia, perivascular macrophages) then other approaches such as transwell filters or chip-based technology could be used. The caveat with those approaches is that the more complex the model, the more difficult data is to interpret. In addition, it can be difficult to control the cellular activation states after isolation of all the cell types alone, which then becomes more problematic when combined. However, an advantage of only using brain endothelial cells is that our assay could be utilized to detect adverse reactions of novel compounds on permeability.

A second limitation is that our model lacks a mimic of blood-flow, such as shear stress, which could alter signaling and the response to stressors. There are ways to include flow in vitro but tend to be less high-throughput or require more specialist technology.

Third is the use of mouse cells as opposed to human cells. In many ways, this criticism has been applied to all research involving mice, and in our opinion sometimes unwarranted. In terms of brain endothelial cell research for compound screening the options are immortalized human brain endothelial cells, frozen primary human cells or differentiated iPSCs. Passing, freezing and immortalizing cells often lead to phenotypes and signaling and functions that deviate from in vivo, particularly for cells as specialist as brain endothelial cells. Currently, the optimal methods for differentiating iPSCs to a brain endothelial versus an epithelial phenotype are still under development, with the caveat the signaling may not exactly correspond to in vivo due to the nature of the differentiation protocols.

Fourth, we focused on permeability. However, as more research is conducted on brain endothelial cells, other functions may be identified that are more proximal to neuronal dysfunction. We do not currently have data to make in depth suggestions on which brain endothelial functions may have a stronger impact on neuron function than permeability, however we do have some speculations. In our previous in vitro study [18], we found transcriptomics profile was markedly different between *APOE3* and *APOE4* brain endothelial cells in baseline conditions, with 1305 differentially expressed genes. The genes fell into broad categories that included metabolism, inflammation, signaling and ion homeostasis. Mediators of all those pathways could impact neuron function through endothelial cell-neuron cross talk, particularly in the metabolic and inflammation categories. Our ongoing studies are focused on identifying ages where there are changes in behavior but not cerebrovascular leakiness to try and dissect potential soluble mediators that could impact neuron function.

Fifth concerns the use of LPS. We used LPS as proof of concept rather than to mimic a stressor found in a specific neurodegenerative disorder. We also used LPS, as there is a link between peripheral inflammation and neurodegenerative disorders, and we have previously shown low dose LPS treatment can exacerbate blood-brain barrier and behavioral dysfunction in an *APOE4* Aβ models [25]. However, there are likely other stressors that are more relevant for neurodegenerative disorders and the signaling induced by LPS is not the same as a specific condition, which limits the value of developed assay. We are planning to test other stressors in our screen including Aβ, oxygen-glucose deprivation, lipids (e.g. oxidized LDL), reactive oxygen species inducers (e.g. homocysteine) and environmental stressors.

Sixth is the applicability of *APOE4* brain endothelial cell dysfunction to neurodegeneration and the specificity of endothelial cell dysfunction to one disease. There is strong evidence that *APOE4* is associated with brain endothelial cell dysfunction in neurodegenerative disorders, however the specific contribution of brain endothelial cell *APOE4* expression is unclear. Therefore, further research is required to delineate the impact of brain endothelial cell *APOE* on cerebrovascular, neuronal and behavioral function which is part of our ongoing research. More generally, brain endothelial cell dysfunction is a feature of multiple neurodegenerative disorders, and therefore whether it is a consequence or a contributing factor to one or all conditions is unclear. Linked to that issue, is that there may be disease specific changes in brain endothelial cell signaling that would be overlooked by focusing on permeability.

Seventh is that we focused on *APOE4* brain endothelial cells rather than conducting a parallel screen with *APOE3* brain endothelial cells. That approach would have enabled us to identify any differences in compound effects between *APOE4* and *APOE3* brain endothelial cells to help establish whether any treatments are specific to *APOE4*. On this point we will present our overarching hypothesis and some supporting data. Our long-term goal is to test the idea that brain endothelial cell *APOE* is protective however *APOE4* is less protective than *APOE3*. Due to the *APOE* genotype specific differences, we consider that *APOE4* brain endothelial cells are more vulnerable to stress-induced dysfunction than *APOE3* brain endothelial cells. Embedded in that concept is that *APOE3* brain endothelial cells are still disrupted by disease relevant stressors through the same pathways as *APOE4*, but to a less extent. Therefore, we propose that brain endothelial cell *APOE4* is a relative loss of positive function rather than a toxic gain of function. In support of that concept, we find that at higher concentrations of LPS, *APOE4* brain endothelial cells show greater TEER disruption than *APOE3* brain endothelial cells (Additional file 4 Supplementary Fig. 4). In addition, SG1-7079, CCT196969, vorinostat and sidenafil protected *APOE3* brain endothelial cells from LPS-induced TEER disruption. There are caveats to our hypothesis, in that there may be some treatments (e.g. that target the structure of apoE) and stressors that end up specific to *APOE4* brain endothelial cells. Our ongoing studies are focused on dissecting the extent that brain endothelial cell *APOE3* and *APOE4* can protect brain endothelial cells and neurons in vivo, including identifying potential pathways. Once we have established *vivo* effects, we will be able to return in vitro to dissect more detailed mechanistic hypothesis.

Eighth surrounds our in vivo study as we did not explore the mechanism of action of our top hits. Any of the compounds could have impacted peripheral inflammation and non-brain endothelial functions in the brain and we did not determine if neuron function and behavior was improved in the model.

## Conclusion

Our data supports the potential of cultured *APOE4*-brain endothelial cells screen to identify compounds that prevent LPS-associated brain endothelial cell dysfunction.

## Supplementary Information

Additional File 1. Supplementary Table 1. Major Resources

Additional File 2. All statistical analysis

Additional File 3. Supplementary Table 2. Total amount/volume of resources

Additional File 4. Supplementary Figures

## Data Availability

The datasets used and/or analyzed during the current study are available from the corresponding author on reasonable request.

## Abbreviations

AD: Alzheimer’s disease
APOE: Apolipoprotein E gene
apoE: Apolipoprotein E protein
AXL: AXL Receptor Tyrosine Kinase
ALK: ALK Receptor Tyrosine Kinase
Aβ: Amyloid beta peptide
BBB: Blood brain barrier
BEC: Brain endothelial cells
BSA: Bovine serum albumin
cAMP: Cyclic Adenosine 3’,5’-Monophosphate
cGMP: Cyclic guanosine monophosphate
CNS: Central nervous system
CSF: Cerebrospinal fluid
CX: Cortex
DMSO: Dimethyl Sulfoxide
E4FAD-: E4FAD-non carrier mice (5xFAD^−/−^/APOE4^+/+^)
EBM-2: Endothelial cell growth medium-2
EGFR: Epidermal growth factor receptor
ELISA: Enzyme-linked immunosorbent assay
eNOS: Endothelial nitric oxide synthase
FDA: Food and drug administration
h: Hours
HDAC: Histone deacetylase
IgG: Immunoglobulin G
JAK: Janus kinase
LPS: Lipopolysaccharide
PBS: Phosphate-buffered saline
PDE: Phosphodiesterase
Raf: Rapidly accelerated fibrosarcoma
MAPK: Mitogen-activated protein kinase
mTOR: Mammalian target of rapamycin
SIL: Didenafil
SDS: Sodium dodecyl sulfate
TEER: Trans endothelial cell electrical resistance
TGFβR: Transforming growth factor beta receptor
TLR4: Toll-like receptor 4
U: Unit
VEGFR: Vascular endothelial growth factor receptor
VC: Vehicle control
