# Supplementary material for: Proof-of-concept study: APOE4 brain endothelial cells as a phenotypic compound screen
Source: Alzheimers Res Ther. 2026 Feb 2;18:54. doi: 10.1186/s13195-026-01960-6 (PMC12964606; doi:10.1186/s13195-026-01960-6)
Supplement: Supplementary file 1 — Additional File 1. Supplementary Table 1. Major Resources [file 13195_2026_1960_MOESM1_ESM.docx]

| **Compound** | **Application** | **Vendor or Source** | **Catalog #** | **[Stock] in DMSO** | **Persistent ID / URL** |
| --- | --- | --- | --- | --- | --- |
| Heparin sodium salt from porcine intestinal mucosa | Isolation | Sigma-Aldrich | H3149-250KU |  | https://www.sigmaaldrich.com/US/en/product/sial/h3149 |
| Puromycin dihydrochloride from Streptomyces alboniger | Isolation | Sigma-Aldrich | P8833-25MG |  | https://www.sigmaaldrich.com/US/en/product/sigma/p8833?srsltid=AfmBOor7hGTlf8IM1UadWc  VCTnMbrjRFNYr9Ti7QcQo8awevf53MQvE6 |
| Gibco™ HBSS, calcium, magnesium, no phenol red | Isolation | Gibco | 14-025-134 |  | https://www.fishersci.com/shop/products/gibco-hbss-calcium-magnesium-no-phenol-red-4/14025134 |
| Bovine Serum Albumin (BSA) Powder--Standard Grade | Isolation | GeminiBio | 700-100P |  | https://store.geminibio.com/s/product/detail/01t4P00000B7qbzQAB |
| Gibco™ HBSS, no calcium, no magnesium, no phenol red | Isolation | Gibco | 14-175-103 |  | https://www.fishersci.com/shop/products/gibco-hbss-without-calcium-magnesium-  phenol-red-4/14175103 |
| DNase vial, PDS kit, 5 vi | Isolation | Worthington Biochemical | LK003172 |  | https://www.worthington-biochem.com/products/deoxyribonuclease-i/manual |
| Papi vial, PDS kit 5vi | Isolation | Worthington Biochemical | LK003178 |  | https://www.worthington-biochem.com/products/papain#product-lines |
| Minimum essential medium eagle, hepes mo, | Isolation | Neta Scientific | SIAL-M7278-6X500ML |  | https://www.netascientific.com/animal-serum/sial-m7278-6x500ml |
| Fibronectin from human plasma | Seeding | Sigma-Aldrich | F0895-5ML |  | https://www.sigmaaldrich.com/US/en/product/sigma/f0895 |
| Collagen from calf skin | Seeding | Sigma-Aldrich | C8919-20ML |  | https://www.sigmaaldrich.com/US/en/product/sigma/c8919 |
| Laminin from Engelbreth-Holm-Swarm murine sarcoma basement membrane | Seeding | Sigma-Aldrich | L2020-1MG |  | https://www.sigmaaldrich.com/US/en/product/sigma/l2020 |
| L-Cysteine | Seeding | Sigma-Aldrich | C7352-25G |  | https://www.sigmaaldrich.com/US/en/product/sigma/c7352?msockid=2826970cad31621237dc8224ac5063ee |
| EGM-2-MV bulletkit | Culture | Fisher Scientific Company LLC | NC9902887 |  | https://www.fishersci.com/shop/products/egm-2-mv-bulletkit/NC9902887 |
| LPS from E. coli O8:K27 (S-form) | Cell treatment | Innaxon Biosciences | IAX-100-006 | 1mg/ml in water (No DMSO) | https://innaxon.co.uk/lps-e-coli-o8k27-s-form-tlrpure-sterile-aqueous-solution-cat-no-iax-100-006-453-p.asp |
| Dimethyl sulfoxide | Compound dissolvent | Thermo Scientific | AAJ66650AD |  | https://www.fishersci.com/shop/products/dimethyl-sulfoxide-  bioreagent-thermo-scientific/AAJ66650AD |
| EHNA, Hydrochloride | Positive control screen | Sigma-Aldrich | 324630 | 10 mg/ml | https://www.sigmaaldrich.com/US/en/product/mm/324630 |
| BAY 60-7550 | Positive control screen | Sigma-Aldrich | SML2311 | 10 mg/ml | https://www.sigmaaldrich.com/US/en/product/sigma/sml2311 |
| Milrinone | Positive control screen | Sigma-Aldrich | M4659 | 10 mg/ml | https://www.sigmaaldrich.com/US/en/product/sigma/m4659?srsltid=AfmBOoq7IClaOkkuCQG9lgNifDL  Ed0DgTEupGjabx3tEvLRinPCd32yd |
| Anagrelide | Positive control screen | Sigma-Aldrich | SML0846 | 5 mg/ml | https://www.sigmaaldrich.com/US/en/product/sigma/sml0846?srsltid=AfmBOoqZQznJakBXRaL7M0  VlnOxlUHkrbrzkA7Bk-veIe1ig96Qhw70Q |
| Vardenafil | Positive control screen | Apexbio | 502240307 | 1 mg/ml | https://www.fishersci.com/shop/products/vardenafil-10mg-2/502240307 |
| Dipyridamole | Positive control screen | Sigma-Aldrich | D9766-1G | 10 mg/ml | https://www.sigmaaldrich.com/US/en/product/sigma/d9766?srsltid=AfmBO  or5g2TMrAKTKDz56_dFrIspHPVB_oEdLho6ATuQcyP2l3Wn_Hu_ |
| Zaprinast | Positive control screen | Sigma-Aldrich | Z0878 | 10 mg/ml | https://www.sigmaaldrich.com/US/en/product/sigma/z0878?srsltid=AfmBOorU--OrEF-YCXAeex5YWG9nHY8OvXYLKpFyVTYSZBn50Z53Qqes |
| BAY 73-6691 | Positive control screen | Sigma-Aldrich | B3561 | 10 mg/ml | https://www.sigmaaldrich.com/US/en/product/sigma/b3561?srsltid=AfmBOoq  LhB75Vu2eMhwgxZJ5ndJeM4dqB2OYnQBJewffGxHY2uXZ-PnY |
| Vesnarinone | Positive control screen | Medchemexpress LLC | HY-15297 | 10 mg/ml | https://www.medchemexpress.com/Vesnarinone.html |
| Cilostazol | Positive control screen | VWR Funding Inc | 101757-854 | 12.5 mg/ml | https://www.avantorsciences.com/us/en/product/NA2230976/cilostazolpletal73963-72-1?isCatNumSearch=true |
| Sildenafil Citrate | Positive control screen | VWR | 101762-766 | 25 mg/ml | https://www.avantorsciences.com/us/en/product/NA2233427/sildenafil-citrate171599-83-0?isCatNumSearch=true |
| 3BDO \| SML1687-5MG | mTOR pathway screen | Sigma-Adrich | SML1687 | 5 mg/ml | https://www.sigmaaldrich.com/US/en/product/sigma/sml1687?srsltid=AfmBOorlQ  Cg9cf_Rmsxu42Uh2Vy_Nds6LYH3quEDXDkOnuyuokm5Aokj |
| NV-5138 (hydrochloride) | mTOR pathway screen | Medchemexpress LLC | 50-225-9319 | 5 mg/ml | https://www.medchemexpress.com/nv-5138-hydrochloride.html |
| MHY1485 | mTOR pathway screen | 18453 | 103547-158 | 5 mg/ml | https://www.avantorsciences.com/us/en/product/NA4602540/mhy1485-9909--powder-326914-06-1-10mg |
| L-Leucine | mTOR pathway screen | Thermo Scientific Chemicals | AAJ62824-22 | 5 mg/ml | https://www.fishersci.com/shop/products/l-leucine-cell-culture-reagent/AAJ6282422 |
| Vorinostat | Hit Validation | VWR Funding Inc | 102992-856 | 10 mg/ml | https://pr.vwr.com/store/product/21641596/n-hydroxy-n-phenyloctanediamide |
| SGI-7079 | In-vivo validation | TARGETMOL CHEMICALS INC | 502576396 | 66.7 mg/ml | https://www.fishersci.com/shop/products/sgi-7079-10mg/502576396 |
| Cayman-Cct196969 | In-vivo validation | Cayman | CAYM-25537-25 | 33.3 mg/ml | https://www.caymanchem.com/product/25537 |
| Cayman-SAHA (Vorinostat) | In-vivo validation | Cayman | CAYM-10009929-100 | 66.7 mg/ml | https://www.caymanchem.com/product/10009929 |
| Tadalafil | In-vivo validation | Sigma-Aldrich | SML1877 | 26.7 mg/ml | https://www.sigmaaldrich.com/US/en/product/sigma/sml1877?srsltid=AfmBOopQx21O_n7S9Tu-TdHWUJT7Nr13jVzTeBz7on9_ZO86cjDAL1iO |
| Cellpro sterile saline 0.9% 1l | In-vivo validation | Alkali Scientific | 104026-756 |  | https://alkalisci.com/cellpro-sterile-saline-0-9-sodium-chloride-usp-sterile-grade/ |
| DMSO LC-MS grade 50ml | In-vivo validation | LIFE TECHNOLOGIES MS | PI85190 |  | https://www.fishersci.com/shop/products/pierce-dimethylsulfoxide-dmso-1/PI85190 |
| BCA Protein assay kit | In-vivo validation | ThermoFisher | 23227 |  | https://www.thermofisher.com/order/catalog/product/23227 |
| Mouse IgG ELISA Kit | In-vivo validation | Immunology Consultants | E-90G |  | https://www.icllab.com/mouse-igg-elisa-kit-e-90g.html |
| Rapamycin | mTOR validation | Avantor | 102515-760 | 5 mg/ml | https://www.avantorsciences.com/us/en/product/NA2574238/rapamycin-from-streptomyces-hygroscopicus-98?isCatNumSearch=true&searchedCatalogNumber=102515-760 |
| Anti-Phospho-4E-BP1 (Ser65) | mTOR validation | Cell Signaling Technology | 9451S | 1:500 | https://www.cellsignal.com/products/primary-antibodies/phospho-4e-bp1-ser65-antibody/9451 |
| Anti-Total 4E-BP1 | mTOR validation | Cell Signaling Technology | 9644T | 1:500 | https://www.cellsignal.com/products/primary-antibodies/4e-bp1-53h11-rabbit-monoclonal-antibody/9644 |
| Anti-GADPH | Western blot loading contro | Cell Signaling Technology | 97166 | 1:1000 | https://www.cellsignal.com/products/primary-antibodies/gapdh-d4c6r-mouse-monoclonal-antibody/97166 |
| Acetyl-Histone 3 (Lys9) | Vorinostat validation | Cell Signaling Technology | 9649 | 1:500 | https://www.cellsignal.com/products/primary-antibodies/acetyl-histone-h3-lys9-c5b11-rabbit-monoclonal-antibody/9649 |
| Total Histone 3 | Vorinostat validation | Cell Signaling Technology | 4243 | 1:500 | https://www.cellsignal.com/products/primary-antibodies/acetyl-histone-h3-lys56-antibody/4243 |

**Supplementary Table 1. Major Resources**
