# Supplementary material for: Proof-of-concept study: APOE4 brain endothelial cells as a phenotypic compound screen
Source: Alzheimers Res Ther. 2026 Feb 2;18:54. doi: 10.1186/s13195-026-01960-6 (PMC12964606; doi:10.1186/s13195-026-01960-6)
Supplement: Supplementary file 3 — Additional File 3. Supplementary Table 2. Total amount/volume of resources [file 13195_2026_1960_MOESM3_ESM.docx]

**Supplementary Table 2. Total amount/volume of resources**
