# Supplementary material for: Proof-of-concept study: APOE4 brain endothelial cells as a phenotypic compound screen
Source: Alzheimers Res Ther. 2026 Feb 2;18:54. doi: 10.1186/s13195-026-01960-6 (PMC12964606; doi:10.1186/s13195-026-01960-6)
Supplement: Supplementary file 4 — Additional File 4. Supplementary Figures [file 13195_2026_1960_MOESM4_ESM.docx]

**
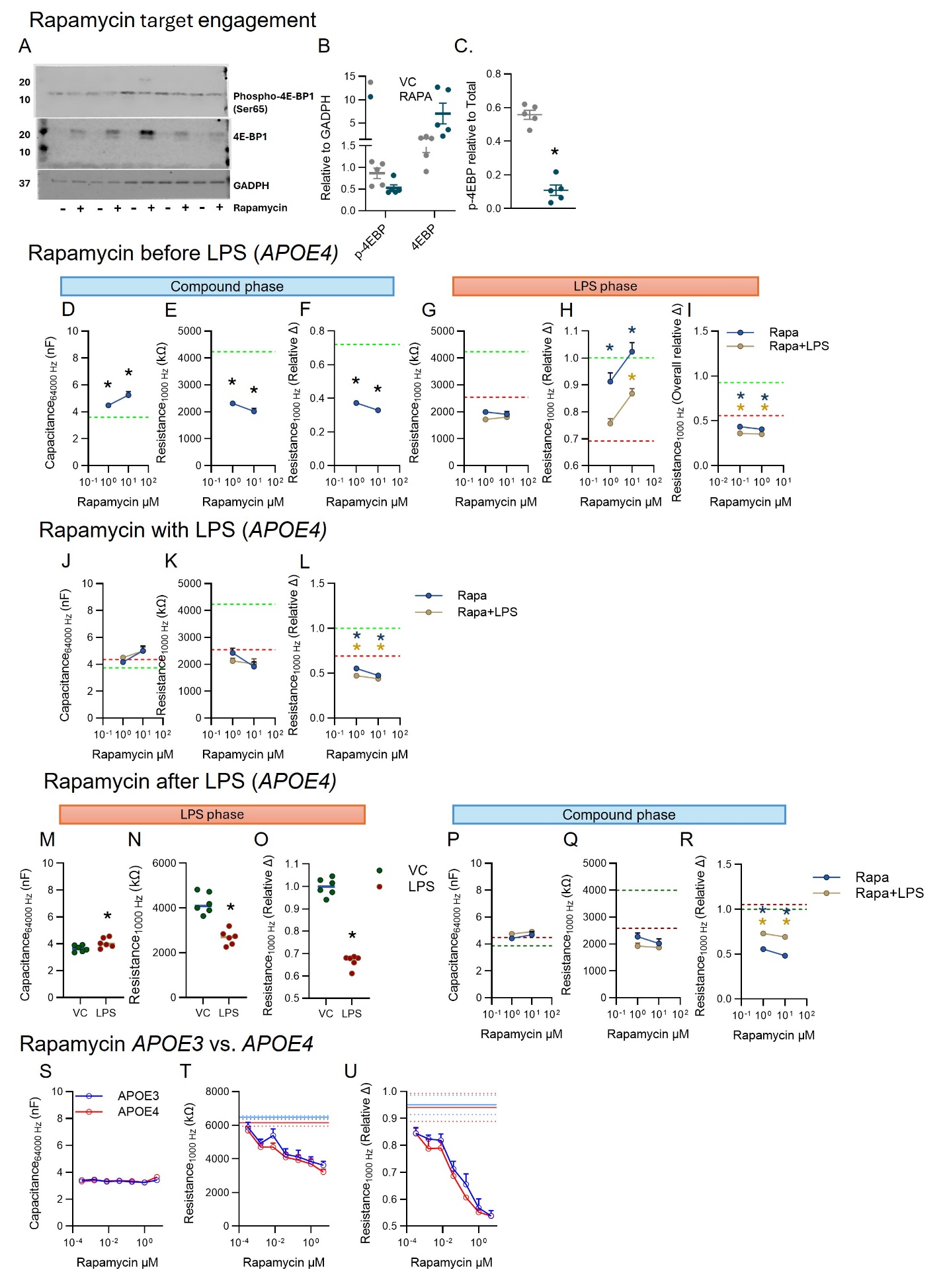
Supplementary Figure 1. mTOR inhibition by Rapamycin disrupts baseline TEER of brain endothelial cells regardless of treatment paradigm and *APOE* genotype.** (**A-I**) *APOE4* brain endothelial cells were treated for 24 h with 1 µM or 10 µM of Rapamycin before LPS treatment, **A-C.** Rapamycin (1 µM) decreased phospho-4EBP1 relative to total 4E-BP1 by 5-fold compared to vehicle **p*<0.0001. **D-F.** 1 µM or 10 µM Rapamycin resulted in slight increase in capacitance and reduced basal levels of TEER (*p<0.05 vs. Vehicle). **G.** LPS treatment did not affect TEER when pre-treated with Rapamycin**. H.** When assessed as relative change, rapamycin showed a false positive protection (*p<0.05 vs. LPS). **I.** Rapamycin only or LPS + Rapamycin treatment (1 µM or 10 µM) reduced overall relative change (before rapamycin treatment) when compared to LPS alone (*p<0.05 vs. LPS)**.** **J-K.** Rapamycin did not affect LPS-induced TEER disruption nor did LPS affect rapamycin-induced TEER disruption when both compounds are added to brain endothelial cells at the same time. **L.** When assessed as relative change, rapamycin treatment with or without LPS reduced TEER compared to LPS alone (*p<0.05 vs. LPS)**.** **M-O.** 18h of LPS treatment disrupted TEER with a slight increase in capacitance (*p<0.05 vs. Vehicle). **P-Q**. Rapamycin treatment did not reverse LPS-induced TEER disruption. **R.** When assessed as relative change, rapamycin treatment with or without LPS reduced TEER compared to LPS alone (*p<0.05 vs. LPS)**.** **S**. Rapamycin treatment did not affect viability in *APOE3-* or *APOE4-*BEC. **T.** Rapamycin reduced TEER disruption (0.0016 – 5 µM) in *APOE3-* or *APOE4-*brain endothelial cells. **U.** Rapamycin reduced relative change in TEER (0.0016 – 5 µM) in *APOE3-* or *APOE4* brain endothelial cells. Upper lines indicate the basal values of *APOE3-* (blue) or *APOE4-*BEC (red). **Data** analyzed by t-test (C, M-O), one-way ANOVA (D-L, P-R) or two way ANOVA (S-U) followed by tukey’s multiple comparisons test comparing each either vehicle or LPS as indicated above. *n*=3-5, when an *n* represents a different isolation. All statistical analysis is provided in Additional File 1

**
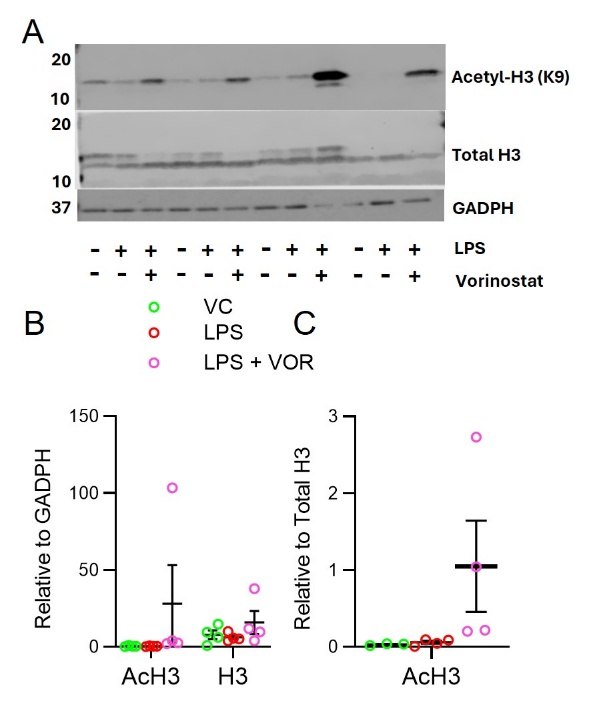
**

**Supplementary Figure 2. Vorinostat treatment increased Histone-3 Acetylation.** *APOE4*-brain endothelial cells were treated with vorinostat (1 µM) or vehicle for 24 hours followed by vehicle or LPS (0.8 µg/ml) for an additional 24 hours. **A-C**. levels of acetyl-H3, GAPDH and total H3 were measured by western blot analysis. Vorinostat treatment resulted in **B.** higher levels of acetyl-H3 in LPS treated cells compared to LPS alone, with no change in total H3 levels resulting in **C.** a higher acetyl-H3/total H3 ratio. Data analyzed by Mann Whitney test **p*<0.05 v.s. LPS. *n*=4, when an *n* represents a different isolation. All statistical analysis is provided in Additional File 1

**
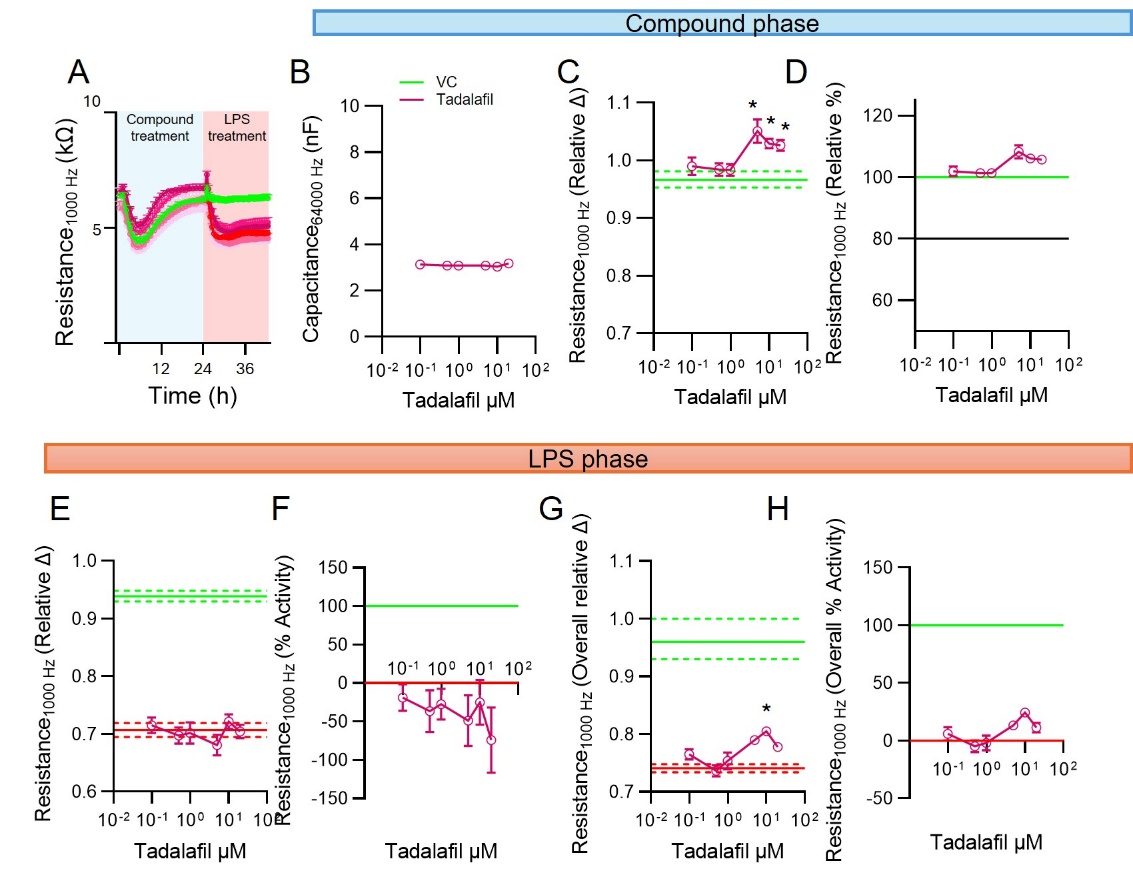
**

**Supplementary Figure 3. Tadalafil raises baseline TEER and partially mitigates LPS-induced disruption in *APOE4* brain endothelial cells. A.** *APOE4*-brain endothelial cells were treated with tadalafil (0.1 - 20 µM) or vehicle for 24 hours followed by vehicle or LPS (0.8 µg/ml) for an additional 24 hours. **B-D.** Compound phase. **B.** Tadalafil was non-toxic and **C-D** raised baseline TEER values at 5 - 10 µM compared to vehicle. **E-H**. LPS phase. **E-F**. When evaluated as relative change or percent activity, tadalafil did not protect against LPS-induced TEER disruption. **G-H**. However, when accounting for the higher baseline levels in the calculation by calculating the relative change using the TEER value before compound addition, 10 µM tadalafil was protective. Data analyzed by 1-way ANOVA followed by Dunnet’s multiple comparisons test comparing each tadalafil concentration to the vehicle (compound phase) or LPS (LPS phase). **p*<0.05 for a given drug concentration compared to vehicle or LPS control *n*=7, when an *n* represents a different isolation. All statistical analysis is provided in Additional File 1


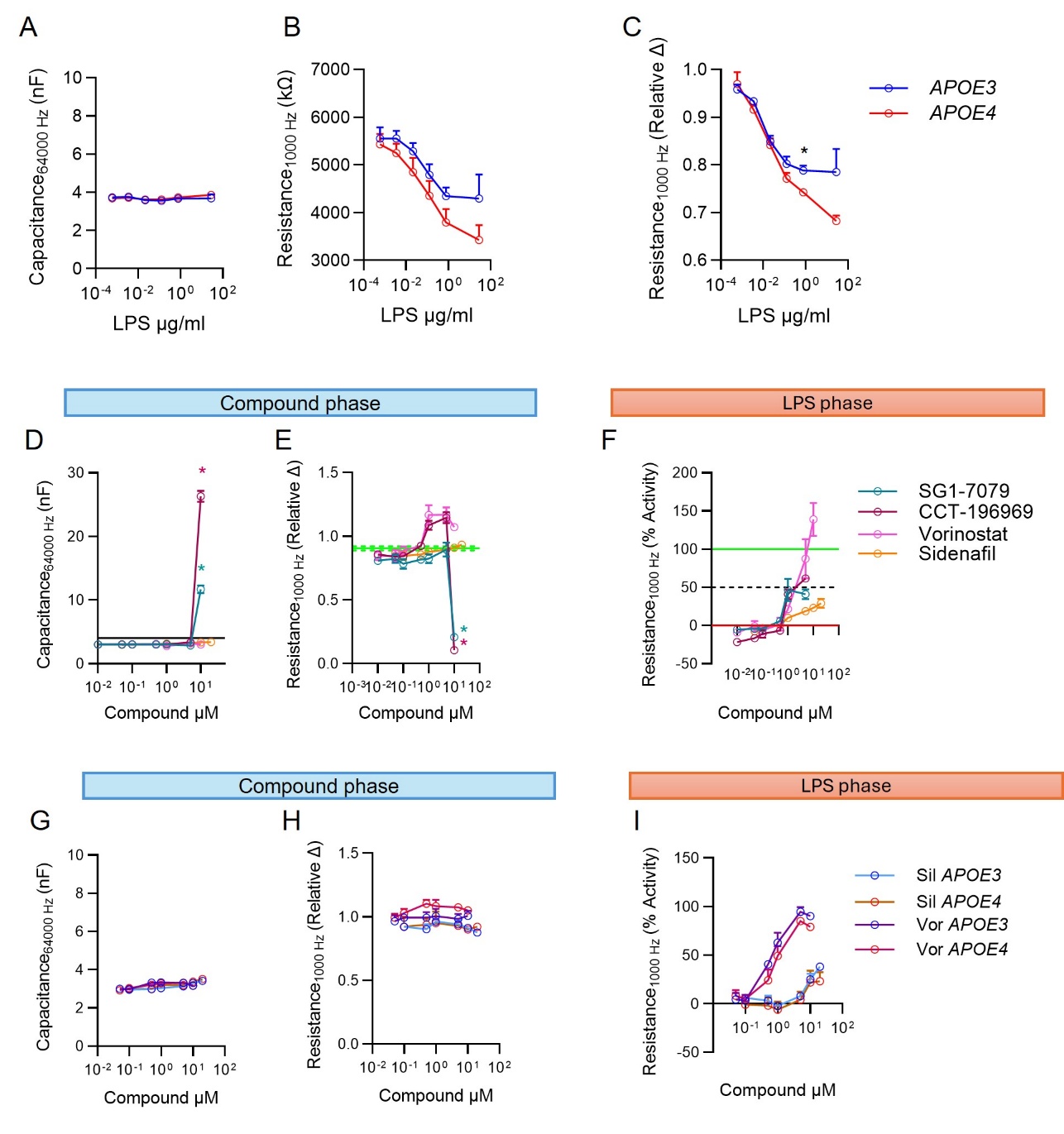
**Supplementary Figure 4. *APOE4* brain endothelial cells are more vulnerable to LPS-induced TEER disruption than *APOE3* brain endothelial cells, which are also protected by SG1-7079, CCT-196969, vorinostat and sildenafil.** **A-C.** *APOE3*- and *APOE4* brain endothelial cells were treated for 24 h with LPS (0.00006-28 µg/ml). For both *APOE* genotypes LPS was **A.** non-toxic and **B-C** lowered relative TEER values at concentrations above 0.021 µg/ml. However, at 0.8 µg/ml the lowering in TEER was greater in *APOE4*- compared to *APOE3*- brain endothelial cells. *p<0.05 *APOE3* vs. *APOE4.* **D-F**. *APOE3* brain endothelial cells were treated with SGI-7079 vorinostat, CCT196969 (0.01 - 10 µM) and sildenafil (0.05-20 µM) for 24 hours followed by 0.8 µg/ml LPS **D-E.** Compound phase. **E.** Toxic compound concentrations were SGI-7079 (10 µM) and CCT196969 (10 µM). CCT196969 (1 and 5 µM) and vorinostat at (1-10 µM) treatment resulted in higher baseline relative TEER values. **G.** LPS Phase. Vorinostat (1-10 µM), CCT-196969 (1-5 µM), SGI-7079 (1-5 µM) and sidenafil (1-20 µM) mitigated LPS-induced TEER reduction. **G-I**. Side by side comparison of sildenafil and vorinostat activity in *APOE3*- and *APOE4*- brain endothelial cells. *APOE3*- and *APOE4* brain endothelial cells were treated with 24 h with vorinostat (0.05 -10 µM) or sildenafil (0.1 - 20 µM) followed by LPS (0.8 µg/ml). **I.** When assessed as relative change sildenafil (10-20 µM) and vorinostat (0.5-10 µM) both mitigated LPS-induced TEER disruption with no differences in the active range or amount of protection at a given concentration between *APOE3* and *APOE4* brain endothelial cells. Data analyzed by matched Mixed-effects model (REML) followed by Dunnet’s multiple comparisons test comparing each LPS concentration to the vehicle (A-C) **p*<0.05 for a given LPS concentration compared to vehicle control or 1-way ANOVA followed by Tukey’s multiple comparisons test (D-I), **p*<0.05 for a given drug concentration compared to vehicle or LPS control. n=3-6 when an *n* represents a different isolation. All statistical analysis is provided in Additional File 1
